# The effect of prenatal balanced energy and protein supplementation on small vulnerable newborn types in low- and middle-income countries: A systematic review and meta-analysis of individual participant data

**DOI:** 10.1371/journal.pmed.1004716

**Published:** 2026-02-17

**Authors:** Dongqing Wang, Uttara Partap, Enju Liu, Janaína Calu Costa, Ilana R. Cliffer, Molin Wang, Sudeer Kumar Nookala, Vishak Subramoney, Brittany Briggs, Imran Ahmed, Alemayehu Argaw, Shabina Ariff, Nita Bhandari, Ranadip Chowdhury, Trenton Dailey-Chwalibóg, Giles T. Hanley-Cook, Lieven Huybregts, Fyezah Jehan, Nancy F. Krebs, Carl Lachat, Dharma S. Manandhar, Elizabeth M. McClure, Sophie E. Moore, Ameer Muhammad, Muhammad Imran Nisar, Andrew M. Prentice, Dominique Roberfroid, Naomi M. Saville, Yasir Shafiq, Bhim P. Shrestha, Bakary Sonko, Sajid Soofi, Sunita Taneja, Laéticia Céline Toe, Wafaie W. Fawzi

**Affiliations:** 1 Department of Global and Community Health, College of Public Health, George Mason University, Fairfax, Virginia, United States of America; 2 Department of Global Health and Population, Harvard T.H. Chan School of Public Health, Harvard University, Boston, Massachusetts, United States of America; 3 Institutional Centers for Clinical and Translational Research, Boston Children’s Hospital, Boston, Massachusetts, United States of America; 4 Division of Gastroenterology, Hepatology and Nutrition, Boston Children’s Hospital, Harvard Medical School, Boston, Massachusetts, United States of America; 5 Department of Epidemiology, Harvard T.H. Chan School of Public Health, Harvard University, Boston, Massachusetts, United States of America; 6 Department of Biostatistics, Harvard T.H. Chan School of Public Health, Harvard University, Boston, Massachusetts, United States of America; 7 Channing Division of Network Medicine, Brigham and Women’s Hospital and Harvard Medical School, Boston, Massachusetts, United States of America; 8 Cytel Inc., India, on behalf of the Gates Foundation, Seattle, Washington, United States of America; 9 DVPL Tech, Dubai, United Arab Emirates; 10 Certara USA, Inc., on behalf of the Gates Foundation, Seattle, Washington, United States of America; 11 Aga Khan University, Karachi, Pakistan; 12 Department of Food Technology, Safety and Health, Faculty of Bioscience Engineering, Ghent University, Ghent, Belgium; 13 Society for Applied Studies, New Delhi, India; 14 Agence de Formation de Recherche et d’Expertise en Santé pour l’Afrique (AFRICSanté), Bobo-Dioulasso, Burkina Faso; 15 Food and Nutrition Division, Food and Agriculture Organization of the United Nations (FAO), Rome, Italy; 16 Nutrition, Diets, and Health Unit, International Food Policy Research Institute, Washington, District of Columbia, United States of America; 17 Department of Pediatrics and Child Health, The Aga Khan University, Karachi, Pakistan; 18 University of Colorado School of Medicine, Aurora, Colorado, United States of America; 19 Mother and Infant Research Activities (MIRA), Kathmandu, Nepal; 20 RTI International, Durham, North Carolina, United States of America; 21 Department of Women & Children’s Health, King’s College London, London, United Kingdom; 22 MRC Unit The Gambia at the London School of Hygiene and Tropical Medicine, Fajara, The Gambia; 23 VITAL Pakistan Trust, Karachi, Pakistan; 24 Faculty of Medicine, University of Namur, Namur, Belgium; 25 Belgian Health Care Knowledge Centre, Brussels, Belgium; 26 Institute for Global Health, University College London, London, United Kingdom; 27 Center of Excellence for Trauma and Emergencies and Community Health Sciences, The Aga Khan University, Karachi, Pakistan; 28 Global Advancement of Infants and Mothers (AIM), Department of Pediatric Newborn Medicine, Brigham and Women’s Hospital, Harvard Medical School, Boston, Massachusetts, United States of America; 29 Harvard Humanitarian Initiative, Harvard T.H. Chan School of Public Health, Harvard University, Boston, Massachusetts, United States of America; 30 Nutrition and Metabolic Diseases Unit, Health Sciences Research Institute (IRSS), Bobo-Dioulasso, Burkina Faso; 31 Department of Nutrition, Harvard T.H. Chan School of Public Health, Harvard University, Boston, Massachusetts, United States of America; The Hospital for Sick Children, CANADA

## Abstract

**Background:**

Small vulnerable newborn (SVN) types, defined by combinations of being born too soon or too small, have distinct determinants, health consequences, and prevention strategies. The effects of prenatal balanced energy and protein (BEP) supplementation on SVN types remain unknown.

**Methods and findings:**

We conducted a systematic review and meta-analysis of individual participant data from eight randomized controlled trials of prenatal BEP supplements (*N* = 10,252, with 5,164 in the BEP arm and 5,088 in the control arm) in low- and middle-income countries were used. The control arms varied across studies and included context-specific standards of care, iron and folic acid supplements, or multiple micronutrient supplements. Newborns were classified into 10 groups through the combinations of preterm birth, small for gestational age (SGA) birth, and low birthweight (LBW), such as term-appropriate-for-gestational-age (AGA)-nonLBW, preterm-SGA-LBW, preterm-large-for-gestational-age-LBW, term-SGA-LBW, preterm-AGA-nonLBW, and other permutations. Newborns were also analyzed using a four-group categorization that included term-nonSGA, preterm-nonSGA, term-SGA, and preterm-SGA. Log-binomial models were used to estimate study-specific risk ratios (RRs), which were pooled using meta-analyses. Subgroup analyses were conducted by maternal age, parity, gestational age at enrollment, early pregnancy body mass index, and maternal anemia status. In the 10-group categorization of SVNs, on average, prenatal BEP supplementation led to a 30% lower risk of preterm-SGA-LBW (RR: 0.70; 95% CI [0.53, 0.91]; *P* = 0.009), a 25% lower risk of preterm-AGA-LBW (RR: 0.75; 95% CI [0.60, 0.93]; *P* = 0.009), and a 20% lower risk of term-SGA-LBW (RR: 0.80; 95% CI [0.72, 0.90]; *P* < 0.001). In the four-group categorization, prenatal BEP supplementation led to a 31% lower risk of preterm-SGA (RR: 0.69; 95% CI [0.52, 0.91]; *P* = 0.008) and a 12% lower risk of term-SGA (RR: 0.88; 95% CI [0.81, 0.96]; *P* = 0.005). The protective effect of prenatal BEP supplementation on preterm-SGA was stronger among multiparous women and women without anemia. The protective effects on all three SVN types under the four-group categorization were stronger among women enrolled before 20 weeks of gestation. The main limitations of the study included the absence of some BEP trials and the small event numbers for some SVN types.

**Conclusions:**

Prenatal BEP supplementation reduces the risk of SVNs to varying extents. Further research is needed to determine the optimal targeting approach for providing BEP supplements to vulnerable pregnant women who are most likely to benefit from the supplementation.

## Author summary

**Why was this study done?:**

- Small vulnerable newborn (SVN) types, defined by combinations of being born too soon or too small, have distinct determinants, health consequences, and prevention strategies.
- The effects of prenatal balanced energy and protein (BEP) supplementation on SVN types are unclear.

**What did the researchers do and find?:**

- Individual participant data from eight randomized controlled trials of prenatal BEP supplements in low- and middle-income countries were used, including data from 10,252 pregnant women.
- The consumption of BEP supplements during pregnancy reduced the risks of numerous SVN types, particularly the types that involve small-for-gestational-age births and the types previously shown to confer a greater risk of neonatal mortality.

**What do these findings mean?:**

- This work underscores the public health benefits of BEP in antenatal care in low- and middle-income countries.
- Further work is warranted to determine the effectiveness, cost-effectiveness, and implementation strategies of prenatal BEP supplementation.
- The main limitations included the absence of some BEP trials and the small event numbers for some SVN types.

## Abstract 3

In a systematic review and individual participant meta-analysis, Dongqing Wang and colleagues investigate the effect of prenatal nutritional supplements on small vulnerable newborns in low- and middle-income countries.Image: https://unsplash.com/photos/person-in-teal-long-sleeve-shirt-lying-on-bed-Jf5WbV0uVpgCredit: Solen Feyissa via unsplash

## Introduction

Low birthweight (LBW), defined as birthweight less than 2,500 g, is the leading cause of neonatal mortality and a strong predictor of adverse child health and nutrition outcomes throughout life [1,2]. Despite progress in reducing LBW, its prevalence remains high, especially in low- and middle-income countries (LMICs) [1]. An estimated 19.8 million live births were LBW in 2020 [3], with over 90% in LMICs [4]. LBW can be attributed to two underlying pathways, namely short pregnancy duration and fetal growth restriction. On the one hand, short pregnancy duration is commonly defined by preterm birth. On the other hand, fetal growth restriction is commonly measured using small for gestational age (SGA) births.

Preterm births, SGA, and LBW have traditionally been evaluated as separate outcomes. Attention has recently been paid to the combinations of these adverse birth outcomes that may have distinct mechanisms, health consequences, and intervention strategies [2,5–8]. Under this emerging framework, preterm birth, SGA, and LBW come together under the unified concept of small vulnerable newborns (SVNs) [2,9]. Of 135 million live births globally in 2020, 21.9 million were SVNs with term-SGA, 11.9 million were preterm-nonSGA, and 1.5 million were preterm-SGA [3]. Term-SGA, preterm-nonSGA, and preterm-SGA accounted for 14.7%, 32.8%, and 7.7% of global neonatal deaths in 2020 [3]. SVNs also have elevated risks of growth faltering, noncommunicable diseases, and compromised learning abilities that jeopardize their achievement of full potential in adulthood [3].

Nutritional supplementation during pregnancy may reduce the risks of some of the SVN types. A recent individual participant data meta-analysis in LMICs showed that prenatal multiple micronutrient supplements (MMS) containing three or more micronutrients (including iron and folic acid) reduced the risk of giving birth to SVN types compared to iron and folic acid (IFA) supplements, with the strongest protective effects observed for SVN types that confer the greatest neonatal mortality risk, such as preterm-SGA, preterm-SGA-LBW, preterm-appropriate-for-gestational-age (AGA)-LBW, and term-SGA-LBW [10]. The analysis highlighted the importance of nutritional supplements in antenatal care and provided additional support to the increasing calls for a switch from IFA supplements to supplements that provide multiple micronutrients, such as MMS tablets, as standard antenatal care [10–12].

Balanced energy and protein (BEP) supplementation is another type of supplement that has gained attention in recent years as a promising nutritional intervention for women and children. BEP supplements refer to ready-to-use or ready-to-be-cooked foods where less than 25% of energy comes from protein content [13]. BEP supplements can be provided to pregnant or lactating women to increase energy and protein intake. BEP supplements are often fortified with essential micronutrients or provided with IFA or MMS. Accumulating evidence, including that from systematic reviews and meta-analyses, shows that prenatal BEP supplements reduce the risks of stillbirths and SGA births and increase birthweight and gestational weight gain [13–18]. However, to our knowledge, the effects of prenatal BEP supplements across SVN types have not been evaluated, which hinders an in-depth understanding of the impact of prenatal BEP on the full spectrum of birth outcomes.

Determining the effects of prenatal BEP supplementation on SVN types will help identify the SVN types that are most affected by BEP supplementation, thereby enhancing our understanding of the beneficial effects of prenatal BEP supplements and providing critical information on the potential scale-up and targeting of BEP supplements among pregnant women. In this study, we pooled individual-level data from randomized controlled trials (RCTs) to examine the impact of prenatal BEP supplementation on SVNs in LMICs.

## Methods

### Data source

We conducted a systematic review and meta-analysis of individual participant data of RCTs of prenatal BEP supplementation in LMICs. We described the design of this analysis previously [18]. We systematically searched the literature using PubMed, Embase, Web of Science, and the Cochrane Library from the inception of each database through June 3, 2021, to identify RCTs of prenatal BEP among pregnant women. The search strategy used for each database is provided in S1–S4 Tables. We also reviewed the references of the identified studies and previous systematic reviews [13–17,19] to identify additional studies of relevance. An updated systematic search was conducted on April 5, 2025, with no additional eligible studies identified.

The inclusion criteria were: (1) RCTs, including both individually randomized or cluster randomized; (2) participants were pregnant at enrollment or enrolled before pregnancy and followed up during pregnancy; (3) studies conducted in a low-income, lower-middle-income, or upper-middle-income economy defined by the World Bank country classification for the 2021 fiscal year; (4) a dietary supplement provided during pregnancy in which less than 25% of the energy is from protein. The supplement could take various forms, such as food rations, beverages, or lipid-based nutrient supplements. When the proportion of energy from protein was not directly reported, we calculated it as grams of protein × 4 kcal ÷ total amount of energy in kcal × 100%; (5) the dietary supplement could be provided alone or in combination with a co-intervention similar across study groups; and (6) at least one study group did not receive BEP supplements. We excluded studies conducted exclusively among women with pre-existing health conditions, such as anemia, diabetes, preeclampsia, or infection with the human immunodeficiency virus. We also excluded studies of small-quantity lipid-based nutrient supplements (SQ-LNS), which provide less than 120 kcal per day; we specifically examined the effects of SQ-LNS on SVNs elsewhere [10].

Two team members independently screened titles and abstracts, with discrepancies resolved by discussion. After the full-text screening, we contacted the corresponding authors of all identified studies to seek collaboration and data contribution. For those willing to participate, we worked with the study teams to pursue appropriate data-sharing agreements. As individual participant data became available, we worked to examine data completeness, map relevant variables, and harmonize the data across studies. This data-sharing and harmonization process was supported by the Knowledge Integration team at the Gates Foundation. Data from 26 studies were sought, of which 11 provided individual participant data for the present analysis. Of these 11 studies, three were further excluded due to the lack of all necessary data to derive SVN types, resulting in eight studies. The characteristics of the eight studies included in the final analysis are shown in Table 1 [20–27]. The characteristics of the 18 non-included studies (15 due to lack of individual participant data and three due to the lack of essential data to derive SVN types) are shown in S5 Table. This study is reported as per the Preferred Reporting Items for Systematic Reviews and Meta-Analyses (PRISMA) guideline (S1 Checklist). The PRISMA flow diagram is shown in S1 Fig.

**Table 1:** Characteristics of the eight studies included in the individual participant data meta-analysis of the effect of prenatal BEP supplements on small vulnerable newborns^1^.

Study	Country	Sample size in analysis	Intervention	Control	Timing of intervention initiation	Daily energy content	Forms of BEP	GA measure
Huybregts (2009)	Burkina Faso	1,041	72 g of MMN-fortified food supplement providing 1.56 MJ (372 kcal) and 14.7 g protein. The food supplement consisted of 33% peanut butter, 32% soy flour, 15% vegetable oil, 20% sugar, and UNIMMAP in powdered form	MMS as the UNIMMAP formulation	Mean gestational age at enrollment around 16 weeks	372 kcal	Lipid-based supplement	Ultrasound (39% conducted in first trimester and 54% in second trimester)
Moore (2012)^2^	The Gambia	666	1) BEP + IFA: A food-based supplement providing a comparable level of iron and folate to the IFA-only arm but with the addition of energy, protein, and lipids. The food supplement provided 746 kcal and 20.8 g protein. 2) BEP + MMS: A micronutrient-fortified, food-based supplement providing comparable levels of micronutrients to the MMS arm and energy, protein, and lipid content. The food supplement provided 746 kcal and 20.8 g protein	1) IFA with 60 mg iron and 400 μg folate, representing the usual standard of care during pregnancy. 2) MMS as the UNIMMAP formulation.	<20 weeks gestation	746 kcal	Lipid-based supplement	Ultrasound conducted around 12 weeks
Saville (2018)^3^	Nepal	2074	PLA + 150 g Super Cereal (fortified wheat soya blend plus 10% sugar) per day, providing 570 kcal, with 17% from protein, and meeting most micronutrient requirements for pregnant women	1) Control; 2) PLA; 3) PLA + cash	>8 weeks gestation	570 kcal	Food ration	LMP
Hambidge (2019)^4^	Guatemala, India, Pakistan	1,233	Small-quantity LNS of 118 kcal and 2.6 g protein. Participants were also provided with an additional daily lipid-based protein-energy supplement if they had a BMI < 20 at any time while receiving the small-quantity LNS or had weight gain in the second or third trimesters of pregnancy less than the Institute of Medicine guidelines; if consumed completely, this additional supplement provided 300 kcal and 11 g protein (∼15% energy) without additional supplemental micronutrients	Standard of care (typically IFA)	12–14 weeks gestation	418 kcal	Lipid-based supplement	Ultrasound conducted in the first trimester
Khan (2021)^3^	Pakistan	46	A monthly ration of 5 kg wheat soya blend plus (165 g/d) was provided to women during pregnancy and for the first 6 months of lactation. A daily ration of supplements provided 633 kcal and 29.1 g protein	Routine standard of care	19% enrolled <12 weeks gestation, 55% enrolled 13–27 weeks gestation, and 25% enrolled ≥28 weeks	633 kcal	Food ration	LMP
Taneja (2022)^5^	India	2009	Interventions in four domains: health, nutrition, psychosocial care and support, and WaSH during preconception, pregnancy, and early childhood. Weekly supplies of locally prepared snacks containing cereal, pulses, soya, oil, sugar, salt, and milk powder (210 kcal, 2 g protein in the second trimester, and 400 kcal, 21 g protein in the third trimester) were provided for daily consumption for women with BMI <25. All women were also given milk (180 ml, 70 kcal, 6 g protein) 6 days a week throughout pregnancy. Women with BMI <18.5 or inadequate GWG were provided one additional hot cooked meal six days a week (500 kcal, 20 g protein) until delivery	Routine antenatal care from governmental or private sources	9–13 weeks gestation	280–970 kcal depending on trimester, BMI, and GWG	Food ration	Ultrasound conducted when two consecutive missed periods were reported
de Kok (2022)	Burkina Faso	1,547	LNS in the form of an energy-dense peanut paste fortified with MMNs. The 72 g daily supplement provided 393 kcal and comprised 36% lipids, 20% protein, and 32% carbohydrates. Protein came from soy (61%), milk (25%), and peanut (15%)	Standard of care (IFA)	<21 weeks gestation	393 kcal	Lipid-based supplement	Ultrasound (63% conducted in first trimester and 37% in second trimester)
Muhammad (2022)^6^	Pakistan	1,636	Approximately 800 kcal/d and 16–21 g of protein in a day as a ready-to-use supplement	Provision of antenatal care, skilled birth attendants, nutrition counseling, and IFA	8 to <19 weeks gestation	800 kcal	Lipid-based supplement	Ultrasound (new pregnancies in catchment area identified using routine surveillance)

^1^ANC, antenatal care; BMI, body mass index; BEP, balanced energy and protein; GA, gestational age; GWG, gestational weight gain; IFA, iron and folic acid; LMP, last menstrual period; LNS, lipid-based nutrient supplements; MMN, multiple micronutrient; MMS, multiple micronutrient supplements; PLA, participatory learning and action women’s groups; UNIMMAP, UNICEF/WHO/United Nations multiple micronutrient supplements for pregnant and lactating women; WaSH, water, sanitation and hygiene.

^2^Randomized controlled trial with a factorial design. Without statistically significant interaction between interventions, the study arms were collapsed based on the provision of prenatal BEP.

^3^Cluster randomized controlled trial.

^4^The study arm that started supplementation from the preconceptional period was excluded as the analysis focused on the effect of prenatal supplements initiated during pregnancy. Data from the Democratic Republic of the Congo were excluded due to a lack of plausible determinations of gestational age using either ultrasound or the last menstrual period.

^5^Randomized controlled trial with a factorial design. The two arms that included preconceptional intervention were removed because the analysis focused on prenatal supplements, and statistical interaction was found between the effects of preconceptional and antenatal intervention on some of the newborn types.

^6^The intervention arm that provided oral azithromycin in addition to BEP and the intervention arm that provided oral nicotinamide and choline in addition to BEP were removed from the analysis, given the additional effects conferred by oral azithromycin and nicotinamide and choline.

### Small vulnerable newborn types

SVNs included all live newborns who were preterm (<37 completed weeks of gestation), born SGA (weight at birth <10th percentile for sex and gestational age based on the INTERGROWTH-21st newborn size standards), or LBW (weight at birth <2,500 g) [2]. We classified newborns using a 10- and a 4-group categorization [2,6,7]. The 10-group categorization grouped newborns through the combinations of birth outcomes in three dimensions: (1) term birth and preterm birth; (2) SGA, AGA, and large for gestational age (LGA); and (3) LBW and not LBW (nonLBW). The permutations across the three dimensions yielded 10 exhaustive and mutually exclusive combinations. The combinations included (1) term-AGA-nonLBW; (2) preterm-SGA-LBW; (3) preterm-AGA-LBW; (4) preterm-LGA-LBW; (5) term-SGA-LBW; (6) term-AGA-LBW; (7) preterm-AGA-nonLBW; (8) preterm-LGA-nonLBW; (9) term-SGA-nonLBW; and (10) term-LGA-nonLBW. Term-AGA-nonLBW was considered the reference type, whereas the other nine were considered the SVN types. Term-LGA-nonLBW does not entail smallness in the dimension of size or gestational age. However, as LGA is associated with increased risks of birth complications and newborn morbidity [28], we considered the term-LGA-nonLBW type in the analysis as a vulnerable newborn type. The four-group categorization classified newborns into four types: (1) term-nonSGA (nonSGA included AGA and LGA); (2) preterm-nonSGA; (3) term-SGA; and (4) preterm-SGA. The term-nonSGA group was considered the reference type.

### Meta-analyses of intervention effects

We used a two-stage analytical approach to obtain study-specific estimates, which were then combined using fixed-effect and random-effects meta-analytical models [29]. We used the estimates from the random-effects estimates as the primary estimates in reporting and interpretation, given the heterogeneity in the interventions provided and the study populations covered. We used the two-stage approach to maximally account for differences in study designs (e.g., cluster randomization and factorial designs), derive study-specific results that facilitate the evaluation of heterogeneity across studies, and to maximally account for the covariates available in each study.

Within each study, we used log-binomial models to estimate the effects of prenatal BEP supplementation on SVN types. We calculated risk ratios (RRs) and 95% confidence intervals (CIs) comparing prenatal BEP with control arms. Log-binomial models allow for the direct estimation of RRs [30,31]. Compared to logistic models, log-binomial models are especially advantageous because odds ratios from logistic models are not valid approximations for RRs for outcomes with high incidence (e.g., greater than 10%), as was the case for many SVN types [30,31]. We used Poisson models with robust variance estimation to handle the model convergence issue known to occasionally occur with the log-binomial models [32]. We assessed the goodness-of-fit of the log-binomial and modified Poisson models using Pearson chi-squared statistics and deviance residuals. For cluster RCTs, we used modified Poisson models with cluster-robust standard errors [33]. For studies with a factorial design, we examined the statistical interaction between prenatal BEP supplementation and the additional intervention by including a product term between the two interventions. We evaluated the significance of the interaction using likelihood ratio tests. In the absence of statistical interaction, defined as the *P* value for the product term being less than 0.10, we collapsed study arms based on whether prenatal BEP supplements were provided. We conducted intention-to-treat analyses using the randomly assigned treatment assignment as the independent variable.

Missing data were handled using complete case analysis. The percentage of participants missing gestational age data was 7.4%, the percentage of participants missing birthweight data was 38.0%, and the percentage of participants missing infant sex data was 11.0% (S6 Table). We used funnel plots to assess the presence of publication bias when there were at least five studies included in the analysis. We conducted the study-specific analyses using SAS 9.4 (SAS Institute, Cary, North Carolina) and the meta-analyses using Comprehensive Meta-Analysis Software Version 4 (Biostat, Englewood, NJ). Analyses were conducted using a two-sided *α* level of 0.05.

### Sensitivity and subgroup analyses

We used exploratory subgroup analyses to examine effect modification for the four-group categorization by: (1) maternal age (<20 years, 20–29 years, and ≥30 years); (2) parity (0 and ≥1); (3) gestational age at study enrollment (<20 weeks and ≥20 weeks); (4) early-pregnancy body mass index (BMI) (underweight [BMI < 18.5 kg/m^2^], normal weight [BMI: 18.5 kg/m^2^ to < 25.0 kg/m^2^], and overweight or obesity [BMI ≥ 25.0 kg/m^2^]); and (5) maternal anemia at baseline (no anemia: hemoglobin concentration ≥11.0 g/dL; mild anemia: hemoglobin concentration ≥10.0 and <11.0 g/dL; moderate to severe anemia: hemoglobin concentration <10.0 g/dL; only the hemoglobin concentrations collected during the first two trimesters of pregnancy were used) [34]. We also conducted stratified analyses for the four-group categorization by study-level characteristics, including: (1) energy content of the supplementation (250 to <500 kcal/d, or 500 to <1,000); (2) percentage of energy from protein (10% to <15%, or 15% to 20%); (3) forms of the BEP supplement (food rations or lipid-based supplement); and (4) the control group (IFA or MMS).

In a sensitivity analysis, we restricted the analysis to the six studies with ultrasound-based measures of gestational age. In another sensitivity analysis, we adjusted for covariates including maternal age, maternal years of education, parity, gestational age at enrollment, maternal height, pre-pregnancy or early-pregnancy BMI, and hemoglobin concentration at enrollment, all as continuous variables. The availability of these covariates varied across studies, and the covariates available in the study were adjusted. Results from this sensitivity analysis were similar to those from the primary analyses. Therefore, we used complete case analysis without covariate adjustment as the primary approach given the randomized designs that minimized confounding through random intervention allocation. The sensitivity analysis that adjusted for covariates was dependent on covariate availability in the included studies, so residual confounding could not be completely ruled out. However, given that randomized designs of the original studies as well as the similar findings with and without covariate adjustments, the concern of residual confounding was minimal. In another sensitivity analysis, we used the Hartung–Knapp–Sidik–Jonkman method to adjust the variance estimates for the number of studies included in the meta-analyses [35,36].

### Assessment of risk of bias

For individually randomized RCTs, we assessed the risk of bias (ROB) using the ROB-2 tool [37]. For cluster RCTs, we used the ROB-2 tool for cluster-randomized studies [38].

### Ethics considerations

The Harvard T.H. Chan School of Public Health Institutional Review Board determined this secondary analysis of existing data was not human participants research because all data had been deidentified prior to receipt, and no new data collection or human–participant interaction was involved. Informed consent was, therefore, not considered applicable. All individual studies were approved by their respective ethics committees.

## Results

Eight studies were included in the pooled analysis (Table 1) [20–27], including two studies conducted in Burkina Faso [20,26], one in Nepal [22], two in Pakistan [24,27], one in The Gambia [21], one in India [25], and one multi-country study with data from Guatemala, India, and Pakistan [23]. Two of the included studies [22,24] were cluster RCTs, and the other six used individual randomization. Five studies [20,21,23,26,27] used lipid-based supplements as BEP vehicles, and the other three [22,24,25] provided BEP in the form of food rations. The daily energy content of the BEP supplements ranged from 280 to 800 kcal. The control groups varied across studies and included context-specific standards of care, IFA supplements, MMS tablets, or antenatal counseling. The analysis included 10,252 participants, with 5,164 in the BEP arm and 5,088 in the control arm.

Term-AGA-nonLBW was the most common newborn type based on the 10-group categorization, accounting for 51% of the overall sample (Table 2). Common SVN types under the 10-group categorization included term-SGA-nonLBW (16%), term-SGA-LBW (12%), preterm-LGA-nonLBW (7%), and preterm-AGA-nonLBW (5%). For the four-group categorization, the analytical sample included 53% term-nonSGA, 28% term-SGA, 17% preterm-nonSGA, and 2% preterm-SGA. Overall, 19% of the births in the analytical sample had LBW, ranging from 10% to 27% across studies.

**Table 2:** Ten- and four-group categorization of newborn types for the analysis of prenatal balanced energy and protein supplements on small vulnerable newborns^1^.

Newborn type	Total	Control	BEP
	*N* = 10,252	*N* = 5,088	*N* = 5,164
Ten-group categorization			
Term^2^-AGA^3^-nonLBW^4^	5,214 (50.86)	2,493 (49.00)	2,721 (52.69)
Term-SGA^5^-nonLBW	1,660 (16.19)	905 (17.79)	755 (14.62)
Term-SGA-LBW^6^	1,214 (11.84)	654 (12.85)	560 (10.84)
Preterm-LGA-nonLBW	706 (6.89)	310 (6.09)	396 (7.67)
Preterm-AGA-nonLBW	513 (5.00)	248 (4.87)	265 (5.13)
Preterm-AGA-LBW	426 (4.16)	218 (4.28)	208 (4.03)
Term-LGA^7^-nonLBW	225 (2.19)	111 (2.18)	114 (2.21)
Preterm^8^-SGA-LBW	161 (1.57)	89 (1.75)	72 (1.39)
Preterm-LGA-LBW	91 (0.89)	41 (0.81)	50 (0.97)
Term-AGA-LBW	42 (0.41)	19 (0.37)	23 (0.45)
Four-group categorization			
Term-nonSGA	5,481 (53.46)	2,623 (51.55)	2,858 (55.34)
Term-SGA	2,874 (28.03)	1,559 (30.64)	1,315 (25.46)
Preterm-nonSGA	1,736 (16.93)	817 (16.06)	919 (17.80)
Preterm-SGA	161 (1.57)	89 (1.75)	72 (1.39)

^1^Values are counts (percentages). The percentages in each column may not add up exactly to 100% due to rounding. AGA, appropriate for gestational age; LBW, low birthweight; LGA, large for gestational age; BEP, balanced energy and protein; nonLBW, not low birthweight; SGA, small for gestational age.

^2^Term birth is defined as gestational age at birth of 37 completed weeks or above.

^3^Appropriate for gestational age is defined as birthweight between 10th and 90th percentiles for sex and gestational age based on the INTERGROWTH-21st newborn size standards.

^4^Non-low-birthweight is defined as a birthweight of 2,500 g or greater.

^5^Small for gestational age is defined as birthweight less than the 10th percentile for sex and gestational age based on the INTERGROWTH-21st newborn size standards.

^6^Low birthweight is defined as a birthweight less than 2,500 g.

^7^Large for gestational age is defined as birthweight larger than the 90th percentile for sex and gestational age based on the INTERGROWTH-21st newborn size standards.

^8^Preterm birth is defined as gestational age at birth less than 37 completed weeks.

Based on the random-effects models (Table 3), compared to control, prenatal BEP supplementation, on average, led to a 30% lower risk of preterm-SGA-LBW (RR: 0.70; 95% CI [0.53, 0.91]; *P* = 0.009), a 25% lower risk of preterm-AGA-LBW (RR: 0.75; 95% CI [0.60, 0.93]; *P* = 0.009), and a 20% lower risk of term-SGA-LBW (RR: 0.80; 95% CI [0.72, 0.90]; *P* < 0.001). Prenatal BEP did not significantly reduce the risks of other SVN types in the 10-group categorization based on the random-effects models. In the four-group categorization, prenatal BEP supplementation led to a 31% lower risk of preterm-SGA (RR: 0.69; 95% CI [0.52, 0.91]; *P* = 0.008) and a 12% lower risk of term-SGA (RR: 0.88; 95% CI [0.81, 0.96]; *P* = 0.005) (Table 4). Prenatal BEP did not significantly reduce preterm-nonSGA in the random-effects model (RR: 0.91; 95% CI [0.79, 1.04]). No publication bias was detected based on the funnel plots and the Egger’s tests (S2–S12 Figs).

**Table 3:** Effects of prenatal balanced energy and protein supplements on newborn types based on the 10-group categorization^1^.

	Newborn types based on the 10-group categorization									
	Term-AGA-nonLBW	Preterm-SGA-LBW	Preterm-AGA-LBW	Preterm-LGA-LBW	Term-SGA-LBW	Term-AGA-LBW	Preterm-AGA-nonLBW	Preterm-LGA-nonLBW	Term-SGA-nonLBW	Term-LGA-nonLBW
	RR (95% CI)	RR (95% CI)	RR (95% CI)	RR (95% CI)	RR (95% CI)	RR (95% CI)	RR (95% CI)	RR (95% CI)	RR (95% CI)	RR (95% CI)
Number of studies		6	7	5	8	4	7	8	8	7
Huybregts (2009)	Reference	0.64 (0.23, 1.77)	0.98 (0.61, 1.57)	2.49 (0.67, 9.31)	0.68 (0.44, 1.05)	1.89 (0.17, 20.76)	0.78 (0.42, 1.45)	2.40 (1.08, 5.34)	0.99 (0.75, 1.31)	0.32 (0.07, 1.58)
Moore (2012)	Reference	Low events (*n* = 1)	1.01 (0.21, 4.97)	Zero events	1.14 (0.70, 1.85)	Low events (*n* = 1)	Low events (*n* = 1)	0.51 (0.05, 5.58)	1.07 (0.82, 1.38)	1.98 (0.69, 5.70)
Saville (2018)^2^	Reference	1.08 (0.46, 2.51)	1.13 (0.64, 1.99)	1.55 (0.52, 4.64)	1.04 (0.75, 1.44)	Low events (*n* = 4)	1.23 (0.82, 1.86)	1.02 (0.64, 1.63)	1.01 (0.84, 1.20)	0.78 (0.45, 1.37)
Hambidge (2019)^3^	Reference	0.76 (0.60, 0.97)	0.80 (0.47, 1.37)	0.85 (0.78, 0.93)	0.77 (0.68, 0.87)	2.11 (0.54, 8.31)	0.83 (0.43, 1.59)	0.71 (0.50, 1.01)	0.97 (0.90, 1.04)	Low events (*n* = 3)
Khan (2021)^2^	Reference	Low events (*n* = 3)	Zero events	Zero events	0.22 (0.03, 1.41)	Zero events	0.27 (0.03, 2.57)	0.67 (0.05, 8.13)	0.57 (0.16, 2.06)	0.67 (0.06, 7.38)
Taneja (2022)	Reference	0.37 (0.21, 0.67)	0.51 (0.35, 0.74)	Zero events	0.72 (0.58, 0.89)	1.52 (0.46, 5.02)	0.85 (0.56, 1.28)	0.38 (0.07, 2.09)	0.63 (0.51, 0.78)	1.44 (0.70, 2.97)
de Kok (2022)	Reference	0.82 (0.28, 2.43)	0.67 (0.37, 1.22)	0.16 (0.05, 0.53)	0.74 (0.49, 1.12)	Low events (*n* = 2)	0.94 (0.70, 1.27)	0.93 (0.77, 1.13)	0.92 (0.71, 1.19)	1.23 (0.70, 2.16)
Muhammad (2022)	Reference	0.81 (0.36, 1.80)	0.71 (0.50, 1.03)	1.31 (0.58, 2.93)	0.89 (0.67, 1.18)	0.52 (0.17, 1.56)	0.93 (0.61, 1.41)	1.03 (0.79, 1.35)	0.82 (0.60, 1.11)	2.32 (0.83, 6.51)
Pooled, fixed-effect	Reference	0.72 (0.59, 0.87)	0.73 (0.61, 0.89)	0.86 (0.79, 0.93)	0.79 (0.73, 0.87)	1.13 (0.58, 2.20)	0.94 (0.79, 1.11)	0.94 (0.83, 1.08)	0.94 (0.88, 0.99)	1.15 (0.84, 1.56)
Pooled, random-effects	Reference	0.70 (0.53, 0.91)	0.75 (0.60, 0.93)	0.93 (0.50, 1.73)	0.80 (0.72, 0.90)	1.13 (0.57, 2.25)	0.94 (0.79, 1.11)	0.95 (0.78, 1.15)	0.90 (0.80, 1.02)	1.17 (0.81, 1.69)
Heterogeneity										
*I*^2^		18.19%	21.99%	67.24%	21.41%	4.00%	0.00%	28.24%	58.42%	22.28%
*P* for heterogeneity		0.30	0.26	0.016	0.26	0.37	0.74	0.20	0.018	0.26

^1^Values are risk ratios and 95% confidence intervals comparing prenatal BEP supplementation with control, computed using log-binomial or modified Poisson models. Estimates are not available for models marked with zero events. Estimates are also not available for models marked with low events (with the number of outcome events shown in parentheses) due to failure of model convergence. AGA, appropriate for gestational age; BEP, balanced energy and protein; CI, confidence interval; LBW, low birthweight; LGA, large for gestational age; nonLBW, not low birthweight; RR, risk ratio; SGA, small for gestational age.

^2^For cluster-randomized trials, Poisson models with cluster-robust standard errors were used. In Saville, 2018, the number of clusters included in the analysis was 76, with an average cluster size of 27, and an intraclass correlation coefficient of. In Khan, 2021, the number of clusters included in the analysis was 11, with an average cluster size of 4.2, and an intraclass correlation coefficient of.

^3^The study arm that started supplementation from the preconceptional period was excluded as the analysis focused on the effect of prenatal supplementation initiated during pregnancy. Poisson models with cluster-robust standard errors were used to account for country.

**Table 4:** Effects of prenatal balanced energy and protein supplements on newborn types based on the four-group categorization^1^.

	Newborn types based on the four-group categorization			
	Term-nonSGA	Term-SGA	Preterm-nonSGA	Preterm-SGA
	RR (95% CI)	RR (95% CI)	RR (95% CI)	RR (95% CI)
Number of studies		8	8	6
Huybregts (2009)	Reference	0.90 (0.72, 1.13)	1.15 (0.85, 1.56)	0.65 (0.23, 1.79)
Moore (2012)	Reference	1.06 (0.85, 1.31)	0.67 (0.19, 2.34)	Low events (*n* = 1)
Saville (2018)^2^	Reference	1.03 (0.88, 1.21)	1.14 (0.87, 1.49)	1.11 (0.47, 2.61)
Hambidge (2019)^3^	Reference	0.88 (0.87, 0.90)	0.81 (0.54, 1.20)	0.75 (0.59, 0.95)
Khan (2021)^2^	Reference	0.47 (0.18, 1.25)	0.41 (0.08, 2.08)	Low events (*n* = 3)
Taneja (2022)	Reference	0.71 (0.62, 0.81)	0.65 (0.50, 0.84)	0.37 (0.21, 0.66)
de Kok (2022)	Reference	0.87 (0.71, 1.07)	0.89 (0.78, 1.02)	0.82 (0.28, 2.41)
Muhammad (2022)	Reference	0.87 (0.72, 1.05)	0.95 (0.81, 1.11)	0.79 (0.35, 1.77)
Pooled, fixed-effect	Reference	0.88 (0.87, 0.90)	0.91 (0.84, 0.99)	0.71 (0.58, 0.86)
Pooled, random-effects	Reference	0.88 (0.81, 0.96)	0.91 (0.79, 1.04)	0.69 (0.52, 0.91)
Heterogeneity				
*I*^2^		60.21%	48.27%	19.65%
*P* for heterogeneity		0.014	0.060	0.29

^1^Values are risk ratios and 95% confidence intervals comparing prenatal BEP supplementation with control, computed using log-binomial or modified Poisson models. Estimates are not available for models marked with low events (with the number of outcome events shown in parentheses) due to failure of model convergence. BEP, balanced energy and protein; CI, confidence interval; LGA, large for gestational age; nonSGA, not small for gestational age; RR, risk ratio; SGA, small for gestational age.

^2^For cluster-randomized trials, Poisson models with cluster-robust standard errors were used. In Saville, 2018, the number of clusters included in the analysis was 76, with an average cluster size of 27, and an intraclass correlation coefficient of. In Khan, 2021, the number of clusters included in the analysis was 11, with an average cluster size of 4.2, and an intraclass correlation coefficient of.

^3^The study arm that started supplementation from the preconceptional period was excluded as the analysis focused on the effect of prenatal supplementation initiated during pregnancy. Poisson models with cluster-robust standard errors were used to account for country.

In subgroup analyses (S7 Table), the protective effect of prenatal BEP supplementation on preterm-SGA was stronger among multiparous women (RR: 0.53; 95% CI [0.32, 0.87]) compared to nulliparous women (RR: 1.28; 95% CI [0.70, 2.32]; *P* for interaction = 0.026). The protective effects were stronger among women enrolled before 20 weeks of gestation compared to those enrolled at 20 weeks or later for term-SGA (<20 weeks: RR: 0.88; 95% CI [0.81, 0.96]; ≥20 weeks: RR: 1.12; 95% CI [0.92, 1.36]; *P* for interaction = 0.03), preterm-nonSGA (<20 weeks: RR: 0.87; 95% CI [0.76, 0.98]; ≥20 weeks: RR: 1.55; 95% CI [0.87, 2.76]; *P* for interaction = 0.05), and preterm-SGA (<20 weeks: RR: 0.69; 95% CI [0.57, 0.85]; ≥20 weeks: RR: 6.85; 95% CI [1.21, 38.72]; *P* for interaction = 0.01). The protective effect of prenatal BEP supplementation on preterm-SGA was stronger among women with no anemia (RR: 0.33; 95% CI [0.15, 0.73]) compared to those with mild anemia (RR: 1.08; 95% CI [0.78, 1.49]) or moderate to severe anemia (RR: 1.03; 95% CI [0.35, 3.02]; *P* for interaction = 0.026). Stratified analyses by study-level characteristics did not show significant effect heterogeneity by energy content of the supplements, percentage energy from protein, forms of the BEP supplement, or whether IFA or MMS was the control group (S8 Table). In the sensitivity analysis using ultrasound-based measures of gestation age (six studies), the estimates were similar to those from the primary analyses (S9 and S10 Tables). The inference remained the same when the Hartung–Knapp–Sidik–Jonkman method was used to adjust the variance estimates for the number of studies included in the meta-analyses (S11 Table). All eight studies were assessed to have a low ROB (S12 Table).

## Discussion

In this systematic review and meta-analysis of individual participant data in LMICs, we showed that prenatal BEP supplements reduced the risks of numerous SVN types, particularly the types that involve SGA and the types previously shown to confer a greater risk of neonatal mortality [7]. The protective effects of prenatal BEP supplements on SVNs appear stronger among women initiating the supplements before 20 weeks of gestation compared to those at 20 weeks or later.

A growing body of evidence supports that prenatal BEP supplements reduce the risks of stillbirth and SGA and increase gestational weight gain and birthweight [13–18]. Due to demonstrated evidence of the benefits of BEP supplements on pregnancy outcomes, the World Health Organization recommends providing BEP supplements to pregnant women to reduce the risk of stillbirths and SGA births in undernourished populations [39]. The latest *Lancet* Series on Maternal and Child Undernutrition in 2021 also recommended BEP supplements for women in food-insecure households [40]. A recent review on effective interventions to address maternal and child malnutrition listed maternal BEP supplementation for undernourished women as one of the 10 recommended interventions [41].

We showed that, on average, prenatal BEP supplementation leads to a 30% lower risk of preterm-SGA-LBW, a 25% lower risk of preterm-AGA-LBW, a 20% lower risk of term-SGA-LBW, a 31% lower risk of preterm-SGA, and a 12% lower risk of term-SGA. Therefore, in line with prior research that examined SGA as a standalone outcome [13–15,17], prenatal BEP supplements reduce the risks of all SVNs involving SGA under the 10- and 4-group categorizations, with the possible exception of term-SGA-nonLBW, for which the protective effect was not statistically significant (RR: 0.90; 95% CI [0.80, 1.02]). This finding indicates that the benefits of prenatal BEP supplementation may be primarily through the prevention of fetal growth restriction. The considerable beneficial effect of prenatal BEP on preterm-AGA-LBW also suggests potential benefits of BEP through preventing the more severe form of prematurity that leads to LBW. A multi-country pooled analysis including 238,143 live births in nine LMICs showed that preterm-SGA-LBW, preterm-AGA-LBW, term-SGA-LBW, preterm-SGA, and term-SGA were among the SVN types associated with the greatest risk of neonatal mortality, conferring 10.6, 12.9, 4.9, 10.4, and 2.7 times greater risks of neonatal mortality, respectively, compared to the reference types (i.e., term-AGA-nonLBW and term-nonSGA) [7]. SVNs may also experience increased risks of growth faltering, noncommunicable diseases, and failure to reach their full potential as future adults [3]. The impact of BEP supplements during pregnancy and lactation on longer-term maternal and child outcomes warrants further research.

With a small yet growing body of literature on the impact of prenatal interventions on the emerging outcomes of SVNs, this work enables a nuanced comparison between BEP supplements and other prenatal nutritional supplements. In a recent individual participant data meta-analysis of 14 studies in LMICs, we showed that compared to prenatal IFA, prenatal MMS containing three or more micronutrients reduced the risks of preterm-SGA-LBW by 27%, preterm-AGA-LBW by 18%, and term-SGA-LBW by 9%, preterm-SGA by 29%, and term-SGA by 7% [10]. Therefore, the impact of BEP supplements on these SVNs is stronger than for MMS, especially for preterm-AGA-LBW (25% versus 18%), term-SGA-LBW (20% versus 9%), and term-SGA (12% versus 7%). We previously also showed that prenatal SQ-LNS providing less than 120 kcal/d reduced the risk of preterm-LGA-nonLBW but not the other SVNs [10]. Therefore, with similar comparators, medium-quantity BEP supplements that provided at least 250 kcal/d had notably stronger effects than SQ-LNS. This finding is also in line with the expert consultation at the Gates Foundation, which recommended that the energy content of BEP supplements should be no less than 250 kcal per day [42]. The stronger effects of BEP compared to MMS and SQ-LNS were likely due to the provision of additional protein, essential fatty acids, and the extra energy content. For example, additional energy and protein improve maternal metabolism of glucose and amino acids [43], gestational weight gain [18], and placental growth and function [44], effects that MMS or other micronutrient supplements may not fully achieve.

The subgroup analyses provide critical information on the potentially different effects of prenatal BEP supplements by individual characteristics, thereby informing the potential targeting of BEP supplements. We showed that the benefits of prenatal BEP supplementation on SVN types are stronger among participants initiating supplementation before 20 weeks of gestation than those starting at 20 weeks or later. This finding aligns with the growing evidence supporting initiating nutritional supplements from early pregnancy [45] or preconception [25]. The exploratory subgroup analyses also show evidence of potential modification for preterm-SGA by parity and anemia status. The subgroup analyses support the potential of providing targeted BEP supplements to pregnant women who are most likely to benefit from the supplementation for more impactful and cost-effective intervention delivery [46]. Simulation studies have examined the cost-effectiveness of BEP supplementation compared to other nutritional interventions [47,48]. It is important to highlight that the findings from the present study pertain to general populations that are not exclusively undernourished. Efforts are ongoing to investigate the effectiveness and cost-effectiveness of different targeting strategies of BEP supplements in real-world settings in sub-Saharan Africa [49] and South Asia [50].

This study has several limitations. First, we could not include all BEP studies we identified, with the main reasons being the lack of response from the study teams or their inability to contribute individual-level data. Second, as newborns were partitioned into SVN types, the number of events became small for several analyses. This sparse-data issue in the study-specific estimates may also impact the pooled estimates [51,52]. Meta-analysis remains an important approach to investigating intervention effects on rare outcomes, and the use of individual participant data provided an unprecedented opportunity to derive the SVN types, which are usually not reported in individual studies. Third, studies that relied on the last menstrual period for gestational age estimation may have measurement errors for SGA and preterm birth outcomes. In the sensitivity analysis restricted to studies using ultrasound, the estimates were virtually unchanged from those in the primary analyses, highlighting the robustness of the findings. Fourth, we did not adjust for multiple comparisons, which may increase the probability of type I error for the composite null hypothesis that none of the SVN types were affected by the supplements. After false discovery rate adjustments using the Benjamini–Hochberg, the statistically significant effects of prenatal BEP on the SVNs (preterm-SGA-LBW, preterm-AGA-LBW, term-SGA-LBW, preterm-SGA, term-SGA) remained significant. Fifth, the subgroup analyses were exploratory and underpowered. Further well-powered studies are warranted to examine the differential impact of BEP supplements.

In conclusion, prenatal BEP supplements reduce the risks of numerous SVN types, particularly the types involving SGA and the types that confer greater neonatal mortality risk. This work underscores the public health benefits of BEP in antenatal care in LMICs. Further efforts are needed to determine the effectiveness, cost-effectiveness, and implementation strategies of prenatal BEP supplementation.

## Supporting information

S1 Checklist PRISMA Checklist. Preferred Reporting Items for Systematic Reviews and Meta-Analyses (PRISMA) checklist (doi: https://doi.org/10.1136/bmj.n71). This checklist is licensed under CC BY 4.0. https://creativecommons.org/licenses/by/4.0/.
(DOCX)

S1 Table PubMed search strategy for identifying randomized controlled trials of prenatal balanced energy and protein supplementation among pregnant women in low- and middle-income countries. (DOCX)

S2 Table Embase search strategy for identifying randomized controlled trials of prenatal balanced energy and protein supplementation among pregnant women in low- and middle-income countries. (DOCX)

S3 Table Web of Science search strategy for identifying randomized controlled trials of prenatal balanced energy and protein supplementation among pregnant women in low- and middle-income countries. (DOCX)

S4 Table The Cochrane search strategy for identifying randomized controlled trials of prenatal balanced energy and protein supplementation among pregnant women in low- and middle-income countries. (DOCX)

S5 Table Characteristics of the 18 studies identified but not included in the individual participant data meta-analysis of the effect of prenatal balanced energy and protein supplements on small vulnerable newborn types. (DOCX)

S6 Table Missingness in outcome data in the included trials on balanced energy and protein supplements. (DOCX)

S7 Table Effects of prenatal balanced energy and protein supplements on newborn types based on the four-group categorization by maternal characteristics as potential effect modifiers. (DOCX)

S8 Table Effects of prenatal balanced energy and protein supplements on newborn types based on the four-group categorization by study-level characteristics. (DOCX)

S9 Table Effects of prenatal balanced energy and protein supplements on newborn types based on the 10-group categorization when restricting to studies with ultrasound-based measures of gestational age. (DOCX)

S10 Table Effects of prenatal balanced energy and protein supplements on newborn types based on the four-group categorization when restricting to studies with ultrasound-based measures of gestational age. (DOCX)

S11 Table Comparison of pooled random-effects estimates with and without using the Hartung–Knapp–Sidik–Jonkman method to adjust the variance estimates for the numbers of studies included in the meta-analyses. (DOCX)

S12 Table Risk of bias of the included studies. (DOCX)

S1 Fig PRISMA flow diagram for the individual participant data meta-analysis on the effects of prenatal balanced energy and protein supplements on small vulnerable newborn types in low- and middle-income countries. (DOCX)

S2 Fig Funnel plot for the effect of prenatal balanced energy and protein supplements on the small vulnerable newborn type of term-SGA-nonLBW. (DOCX)

S3 Fig Funnel plot for the effect of prenatal balanced energy and protein supplements on the small vulnerable newborn type of term-SGA-LBW. (DOCX)

S4 Fig Funnel plot for the effect of prenatal balanced energy and protein supplements on the small vulnerable newborn type of preterm-SGA-LBW. (DOCX)

S5 Fig Funnel plot for the effect of prenatal balanced energy and protein supplements on the small vulnerable newborn type of preterm-AGA-nonLBW. (DOCX)

S6 Fig Funnel plot for the effect of prenatal balanced energy and protein supplements on the small vulnerable newborn type of preterm-AGA-LBW. (DOCX)

S7 Fig Funnel plot for the effect of prenatal balanced energy and protein supplements on the small vulnerable newborn type of term-LGA-nonLBW. (DOCX)

S8 Fig Funnel plot for the effect of prenatal balanced energy and protein supplements on the small vulnerable newborn type of preterm-LGA-nonLBW. (DOCX)

S9 Fig Funnel plot for the effect of prenatal balanced energy and protein supplements on the small vulnerable newborn type of preterm-LGA-LBW. (DOCX)

S10 Fig Funnel plot for the effect of prenatal balanced energy and protein supplements on the small vulnerable newborn type of term-SGA. (DOCX)

S11 Fig Funnel plot for the effect of prenatal balanced energy and protein supplements on the small vulnerable newborn type of preterm-nonSGA. (DOCX)

S12 Fig Funnel plot for the effect of prenatal balanced energy and protein supplements on the small vulnerable newborn type of preterm-SGA. (DOCX)

## Abbreviations

AGA: appropriate for gestational age
BEP: balanced energy and protein
BMI: body mass index
CI: confidence interval
IFA: iron and folic acid
LBW: low birthweight
LGA: large for gestational age
LMICs: low- and middle-income countries
MMS: multiple micronutrient supplements
PRISMA: Preferred Reporting Items for Systematic Reviews and Meta-Analyses
RCTs: randomized controlled trials
ROB: risk of bias
RR: risk ratio
SGA: small for gestational age
SQ-LNS: small-quantity lipid-based nutrient supplements
SVN: small vulnerable newborn
