# Supplementary material for: The effect of prenatal balanced energy and protein supplementation on small vulnerable newborn types in low- and middle-income countries: A systematic review and meta-analysis of individual participant data
Source: PLoS Med. 2026 Feb 17;23(2):e1004716. doi: 10.1371/journal.pmed.1004716 (PMC12912696; doi:10.1371/journal.pmed.1004716)
Supplement: S1 Table — (DOCX) [file pmed.1004716.s002.docx]

# **S1 Table.** PubMed search strategy for identifying randomized controlled trials of prenatal balanced energy and protein supplementation among pregnant women in low- and middle-income countries

| Concept | PubMed Search terms |
| --- | --- |
| (1) Pregnancy | "Pregnancy"[Mesh] OR Pregnanc*[tiab] OR Pregnant[tiab] OR prenatal[tiab] OR gestation*[tiab] or antenatal[tiab] OR "Pregnant Women"[Mesh] OR gravid[tiab] OR obstetric[tiab] OR antepartum[tiab] |
| (2) Low- and middle-income countries | "Developing Countries"[MeSH] OR “developing countr*”[tiab] OR “developing nation*”[tiab] OR “less developed countr*”[tiab] OR “less developed nation*”[tiab] OR “third world nation*”[tiab] OR “third world countr*”[tiab] OR “under developed nation*”[tiab] OR “underdeveloped nation*”[tiab] OR “under developed countr*”[tiab] OR “underdeveloped countr*”[tiab] OR “middle income countr*”[tiab] OR “middle-income countr*”[tiab] OR “middle income nation*”[tiab] OR “middle-income nation*”[tiab] OR “low income countr*”[tiab] OR “low-income countr*”[tiab] OR “low income nation*”[tiab] OR “low-income nation*”[tiab] OR “poor countr*”[tiab] OR “poor nation*”[tiab] OR lmic[tiab] OR lmics[tiab] OR "Africa"[MeSH] OR "Asia"[MeSH] OR "South America"[MeSH] OR "Latin America"[MeSH] OR "Central America"[MeSH] OR africa[tiab] OR asia[tiab] OR “south america*”[tiab] OR “latin america*”[tiab] OR “central america*”[tiab] OR Afghanistan*[tiab] OR Albania*[tiab] OR Algeria*[tiab] OR Samoa*[tiab] OR Angola*[tiab] OR Armenia*[tiab] OR Azerbaijan*[tiab] OR Bangladesh*[tiab] OR Bengali[tiab] OR Belarus*[tiab] OR Belize[tiab] OR Benin[tiab] OR Bhutan*[tiab] OR Bolivia*[tiab] OR Bosnia*[tiab] OR Herzegovina*[tiab] OR Botswana*[tiab] OR Brazil*[tiab] OR Bulgaria*[tiab] OR “Burkina Faso”[tiab] OR Burkinabe[tiab] OR Burundi*[tiab] OR “Cabo Verd*”[tiab] OR “Cape Verd*”[tiab] OR Cambodia*[tiab] OR Cameroon*[tiab] OR “Central African*”[tiab] OR Chad*[tiab] OR China[tiab] OR Chinese[tiab] OR Colombia*[tiab] OR Comoros[tiab] OR Congo[tiab] OR “Costa Rica*”[tiab] OR “Cote d'Ivoire”[tiab] OR “Ivory Coast”[tiab] OR Cuba[tiab] OR Cuban[tiab] OR Djibouti[tiab] OR Dominica*[tiab] OR Ecuador[tiab] OR Egypt*[tiab] OR “El Salvador*”[tiab] OR Eritrea*[tiab] OR Ethiopia*[tiab] OR Fiji*[tiab] OR Gabon*[tiab] OR Gambia*[tiab] OR Georgia*[tiab] OR Ghana*[tiab] OR Grenada*[tiab] OR Guatemala*[tiab] OR Guinea*[tiab] OR Guyan*[tiab] OR Haiti*[tiab] OR Hondura*[tiab] OR India[tiab] OR Indian*[tiab] OR Indonesia*[tiab] OR Iran*[tiab] OR Iraq*[tiab] OR Jamaica*[tiab] OR Jordan*[tiab] OR Kazakh*[tiab] OR Kenya*[tiab] OR Kiribati[tiab] OR “People's Republic of Korea”[tiab] OR “North Korea”[tiab] OR Kosovo[tiab] OR Kosovar*[tiab] OR Kyrgyz*[tiab] OR Lao[tiab] OR Laos[tiab] OR Laotian*[tiab] OR Lebanon[tiab] OR Lebanes*[tiab] OR Lesotho[tiab] OR Liberia*[tiab] OR Libya*[tiab] OR Macedonia*[tiab] OR Madagascar*[tiab] OR Malawi*[tiab] OR Malaysia*[tiab] OR Maldives[tiab] OR Mali[tiab] OR “Marshall Island*”[tiab] OR Mauritania*[tiab] OR Mexico[MeSH] OR Mexico[tiab] OR Mexican*[tiab] OR Micronesia*[tiab] OR Moldova*[tiab] OR Mongolia*[tiab] OR Montenegr*[tiab] OR Morocc*[tiab] OR Mozambique[tiab] OR Myanmar[tiab] OR Burmese*[tiab] OR Burma[tiab] OR Namibia*[tiab] OR Nepal*[tiab] OR Nicaragua*[tiab] OR Niger*[tiab] OR Niue[tiab] OR Pakistan*[tiab] OR Paraguay*[tiab] OR Peru*[tiab] OR Philippin*[tiab] OR Rwanda*[tiab] OR “Sao Tome”[tiab] OR Principe[tiab] OR Senegal*[tiab] OR Serbia*[tiab] OR “Sierra Leone*”[tiab] OR “Solomon Island*”[tiab] OR Somalia*[tiab] OR “South Africa*”[tiab] OR “Sri Lanka”[tiab] OR “St Lucia”[tiab] OR “Saint Lucia”[tiab] OR “St Vincent”[tiab] OR “Saint Vincent”[tiab] OR Grenad*[tiab] OR Sudan*[tiab] OR Suriname*[tiab] OR Swaziland*[tiab] OR Eswatini*[tiab] OR Syria*[tiab] OR Tajik*[tiab] OR Tanzania*[tiab] OR Zanzibar[tiab] OR Thai*[tiab] OR Timor*[tiab] OR Togo*[tiab] OR Tonga*[tiab] OR Tunisia*[tiab] OR Turkey[tiab] OR Turkish[tiab] OR Turkmen*[tiab] OR Tuvalu*[tiab] OR Uganda*[tiab] OR Ukrain*[tiab] OR Uzbeki*[tiab] OR Vanuatu*[tiab] OR Venezuela*[tiab] OR Vietnam*[tiab] OR “Viet nam*”[tiab] OR “West Bank”[tiab] OR Gaza*[tiab] OR Palestin*[tiab] OR Yemen*[tiab] OR Zambia*[tiab] OR Zimbabw*[tiab] OR “Western Sahara”[tiab] OR Argentin*[tiab] OR Russia*[tiab] |
| (3) Trials | "Clinical Trials as Topic"[Mesh] OR “Randomized Controlled Trial”[pt] OR Clinical Trial[pt] OR “Controlled Clinical Trial”[pt] OR "randomized controlled trials as topic"[MeSH] OR “controlled trial*”[tiab] OR intervention*[tiab] OR "random allocation"[MeSH] OR random*[tiab] OR trial*[tiab] OR "Clinical Trial Protocols as Topic"[Mesh] OR "Clinical Trial Protocol"[pt] OR "Clinical Study"[pt] OR "Clinical Studies as Topic"[Mesh] OR "Therapeutic Uses"[Mesh] OR "therapeutic use"[Subheading] |
| (4) Balanced energy and protein supplements | ("Energy Intake"[Mesh] OR "Dietary Proteins"[Mesh] OR “protein energy”[tiab] OR “energy protein”[tiab] OR protein*[tiab] OR energy*[tiab]) AND ("Dietary Supplements"[Mesh] OR supplement*[tiab] OR supplementation*[tiab]) |
| (5) Animal studies | Animals[Mesh] NOT (Animals[Mesh] AND Humans[Mesh]) |
| Search strategy | (1) And (2) And (3) And (4) Not (5) |
