# Supplementary material for: The effect of prenatal balanced energy and protein supplementation on small vulnerable newborn types in low- and middle-income countries: A systematic review and meta-analysis of individual participant data
Source: PLoS Med. 2026 Feb 17;23(2):e1004716. doi: 10.1371/journal.pmed.1004716 (PMC12912696; doi:10.1371/journal.pmed.1004716)
Supplement: S2 Table — (DOCX) [file pmed.1004716.s003.docx]

# **S2 Table.** Embase search strategy for identifying randomized controlled trials of prenatal balanced energy and protein supplementation among pregnant women in low- and middle-income countries

| Concept | Embase Search terms |
| --- | --- |
| (1) Pregnancy | 'pregnancy'/exp OR Pregnanc*:ab,ti OR Pregnant:ab,ti OR prenatal:ab,ti OR gestation*:ab,ti or antenatal:ab,ti OR 'pregnant woman'/exp OR gravid:ab,ti OR obstetric:ab,ti OR antepartum:ab,ti |
| (2) Low- and middle-income countries | ‘developing country’/exp OR ‘developing countr*’:ab,ti OR ‘developing nation*’:ab,ti OR ‘less developed countr*’:ab,ti OR ‘less developed nation*’:ab,ti OR ‘third world nation*’:ab,ti OR ‘third world countr*’:ab,ti OR ‘under developed nation*’:ab,ti OR ‘underdeveloped nation*’:ab,ti OR ‘under developed countr*’:ab,ti OR ‘underdeveloped countr*’:ab,ti OR ‘middle income countr*’:ab,ti OR ‘middle-income countr*’:ab,ti OR ‘middle income nation*’:ab,ti OR ‘middle-income nation*’:ab,ti OR ‘low income countr*’:ab,ti OR ‘low-income countr*’:ab,ti OR ‘low income nation*’:ab,ti OR ‘low-income nation*’:ab,ti OR ‘poor countr*’:ab,ti OR ‘poor nation*’:ab,ti OR lmic:ab,ti OR lmics:ab,ti OR ‘Africa’/exp OR ‘Asia’/exp OR ‘South America’/exp OR ‘South and Central America’/exp OR ‘Central America’/exp OR ‘Central American’/exp OR africa:ab,ti OR asia:ab,ti OR ‘south america*’:ab,ti OR ‘latin america*’:ab,ti OR ‘central america*’:ab,ti OR Afghanistan*:ab,ti OR Albania*:ab,ti OR Algeria*:ab,ti OR Samoa*:ab,ti OR Angola*:ab,ti OR Armenia*:ab,ti OR Azerbaijan*:ab,ti OR Bangladesh*:ab,ti OR Bengali:ab,ti OR Belarus*:ab,ti OR Belize:ab,ti OR Benin:ab,ti OR Bhutan*:ab,ti OR Bolivia*:ab,ti OR Bosnia*:ab,ti OR Herzegovina*:ab,ti OR Botswana*:ab,ti OR Brazil*:ab,ti OR Bulgaria*:ab,ti OR ‘Burkina Faso’:ab,ti OR Burkinabe:ab,ti OR Burundi*:ab,ti OR ‘Cabo Verd*’:ab,ti OR ‘Cape Verd*’:ab,ti OR Cambodia*:ab,ti OR Cameroon*:ab,ti OR ‘Central African*’:ab,ti OR Chad*:ab,ti OR China:ab,ti OR Chinese:ab,ti OR Colombia*:ab,ti OR Comoros:ab,ti OR Congo:ab,ti OR ‘Costa Rica*’:ab,ti OR ‘Cote d`Ivoire’:ab,ti OR ‘Ivory Coast’:ab,ti OR Cuba:ab,ti OR Cuban:ab,ti OR Djibouti:ab,ti OR Dominica*:ab,ti OR Ecuador:ab,ti OR Egypt*:ab,ti OR ‘El Salvador*’:ab,ti OR Eritrea*:ab,ti OR Ethiopia*:ab,ti OR Fiji*:ab,ti OR Gabon*:ab,ti OR Gambia*:ab,ti OR Georgia*:ab,ti OR Ghana*:ab,ti OR Grenada*:ab,ti OR Guatemala*:ab,ti OR Guinea*:ab,ti OR Guyan*:ab,ti OR Haiti*:ab,ti OR Hondura*:ab,ti OR India:ab,ti OR Indian*:ab,ti OR Indonesia*:ab,ti OR Iran*:ab,ti OR Iraq*:ab,ti OR Jamaica*:ab,ti OR Jordan*:ab,ti OR Kazakh*:ab,ti OR Kenya*:ab,ti OR Kiribati:ab,ti OR ‘People`s Republic of Korea’:ab,ti OR ‘North Korea’:ab,ti OR Kosovo:ab,ti OR Kosovar*:ab,ti OR Kyrgyz*:ab,ti OR Lao:ab,ti OR Laos:ab,ti OR Laotian*:ab,ti OR Lebanon:ab,ti OR Lebanes*:ab,ti OR Lesotho:ab,ti OR Liberia*:ab,ti OR Libya*:ab,ti OR Macedonia*:ab,ti OR Madagascar*:ab,ti OR Malawi*:ab,ti OR Malaysia*:ab,ti OR Maldives:ab,ti OR Mali:ab,ti OR ‘Marshall Island*’:ab,ti OR Mauritania*:ab,ti OR ‘Mexico’/exp OR Mexico:ab,ti OR Mexican*:ab,ti OR Micronesia*:ab,ti OR Moldova*:ab,ti OR Mongolia*:ab,ti OR Montenegr*:ab,ti OR Morocc*:ab,ti OR Mozambique:ab,ti OR Myanmar:ab,ti OR Burmese*:ab,ti OR Burma:ab,ti OR Namibia*:ab,ti OR Nepal*:ab,ti OR Nicaragua*:ab,ti OR Niger*:ab,ti OR Niue:ab,ti OR Pakistan*:ab,ti OR Paraguay*:ab,ti OR Peru*:ab,ti OR Philippin*:ab,ti OR Rwanda*:ab,ti OR ‘Sao Tome’:ab,ti OR Principe:ab,ti OR Senegal*:ab,ti OR Serbia*:ab,ti OR ‘Sierra Leone*’:ab,ti OR ‘Solomon Island*’:ab,ti OR Somalia*:ab,ti OR ‘South Africa*’:ab,ti OR ‘Sri Lanka’:ab,ti OR ‘St Lucia’:ab,ti OR ‘Saint Lucia’:ab,ti OR ‘St Vincent’:ab,ti OR ‘Saint Vincent’:ab,ti OR Grenad*:ab,ti OR Sudan*:ab,ti OR Suriname*:ab,ti OR Swaziland*:ab,ti OR Eswatini*:ab,ti OR Syria*:ab,ti OR Tajik*:ab,ti OR Tanzania*:ab,ti OR Zanzibar:ab,ti OR Thai*:ab,ti OR Timor*:ab,ti OR Togo*:ab,ti OR Tonga*:ab,ti OR Tunisia*:ab,ti OR Turkey:ab,ti OR Turkish:ab,ti OR Turkmen*:ab,ti OR Tuvalu*:ab,ti OR Uganda*:ab,ti OR Ukrain*:ab,ti OR Uzbeki*:ab,ti OR Vanuatu*:ab,ti OR Venezuela*:ab,ti OR Vietnam*:ab,ti OR ‘Viet nam*’:ab,ti OR ‘West Bank’:ab,ti OR Gaza*:ab,ti OR Palestin*:ab,ti OR Yemen*:ab,ti OR Zambia*:ab,ti OR Zimbabw*:ab,ti OR ‘Western Sahara’:ab,ti OR Argentin*:ab,ti OR Russia*:ab,ti |
| (3) Trials | 'clinical trial'/exp OR 'randomized controlled trial'/exp OR 'clinical trial (topic)'/exp OR 'controlled clinical trial'/exp OR 'randomized controlled trial (topic)'/exp OR ‘controlled trial*’:ab,ti OR intervention*:ab,ti OR 'randomization'/exp OR random*:ab,ti OR trial*:ab,ti OR 'clinical trial protocol'/exp OR 'clinical study'/exp OR 'drug therapy'/exp |
| (4) Balanced energy and protein supplements | ('caloric intake'/exp OR 'protein intake'/exp OR ‘protein energy’:ab,ti OR ‘energy protein’:ab,ti OR protein*:ab,ti OR energy*:ab,ti) AND ('supplementation'/exp OR 'vitamin supplementation'/exp OR 'dietary supplement'/exp OR 'food assistance'/exp OR supplement*:ab,ti OR supplementation*:ab,ti) |
| (5) Animal studies | 'animal'/exp NOT ('animal'/exp AND 'human'/exp) |
| Search strategy | (1) And (2) And (3) And (4) Not (5) |
