# Supplementary material for: The effect of prenatal balanced energy and protein supplementation on small vulnerable newborn types in low- and middle-income countries: A systematic review and meta-analysis of individual participant data
Source: PLoS Med. 2026 Feb 17;23(2):e1004716. doi: 10.1371/journal.pmed.1004716 (PMC12912696; doi:10.1371/journal.pmed.1004716)
Supplement: S3 Table — (DOCX) [file pmed.1004716.s004.docx]

# **S3 Table.** Web of Science search strategy for identifying randomized controlled trials of prenatal balanced energy and protein supplementation among pregnant women in low- and middle-income countries

| Concept | Web of Science Search terms |
| --- | --- |
| (1) Pregnancy | TS=Pregnanc* OR TS=Pregnant OR TS=prenatal OR TS=gestation* OR TS=antenatal OR TS=gravid OR TS=obstetric OR TS=antepartum |
| (2) Low- and middle-income countries | TS=“developing countr*” OR TS=“developing nation*” OR TS=“less developed countr*” OR TS=“less developed nation*” OR TS=“third world nation*” OR TS=“third world countr*” OR TS=“under developed nation*” OR TS=“underdeveloped nation*” OR TS=“under developed countr*” OR TS=“underdeveloped countr*” OR TS=“middle income countr*” OR TS=“middle-income countr*” OR TS=“middle income nation*” OR TS=“middle-income nation*” OR TS=“low income countr*” OR TS=“low-income countr*” OR TS=“low income nation*” OR TS=“low-income nation*” OR TS=“poor countr*” OR TS=“poor nation*” OR TS=LMIC OR TS=LMICs OR TS=Africa* OR TS=Asia* OR TS=“south america*” OR TS=“latin america*” OR TS=“central america*” OR TS=Afghanistan* OR TS=Albania* OR TS=Algeria* OR TS=Samoa* OR TS=Angola* OR TS=Armenia* OR TS=Azerbaijan* OR TS=Bangladesh* OR TS=Bengali OR TS=Belarus* OR TS=Belize OR TS=Benin OR TS=Bhutan* OR TS=Bolivia* OR TS=Bosnia* OR TS=Herzegovina* OR TS=Botswana* OR TS=Brazil* OR TS=Bulgaria* OR TS=“Burkina Faso” OR TS=Burkinabe OR TS=Burundi* OR TS=“Cabo Verd*” OR TS=“Cape Verd*” OR TS=Cambodia* OR TS=Cameroon* OR TS=“Central African*” OR TS=Chad* OR TS=China OR TS=Chinese OR TS=Colombia* OR TS=Comoros OR TS=Congo OR TS=“Costa Rica*” OR TS=“Cote d’Ivoire” OR TS=“Ivory Coast” OR TS=Cuba OR TS=Cuban OR TS=Djibouti OR TS=Dominica* OR TS=Ecuador OR TS=Egypt* OR TS=“El Salvador*” OR TS=Eritrea* OR TS=Ethiopia* OR TS=Fiji* OR TS=Gabon* OR TS=Gambia* OR TS=Georgia* OR TS=Ghana* OR TS=Grenada* OR TS=Guatemala* OR TS=Guinea* OR TS=Guyan* OR TS=Haiti* OR TS=Hondura* OR TS=India OR TS=Indian* OR TS=Indonesia* OR TS=Iran* OR TS=Iraq* OR TS=Jamaica* OR TS=Jordan* OR TS=Kazakh* OR TS=Kenya* OR TS=Kiribati OR TS=“People’s Republic of Korea” OR TS=“North Korea” OR TS=Kosovo OR TS=Kosovar* OR TS=Kyrgyz* OR TS=Lao OR TS=Laos OR TS=Laotian* OR TS=Lebanon OR TS=Lebanes* OR TS=Lesotho OR TS=Liberia* OR TS=Libya* OR TS=Macedonia* OR TS=Madagascar* OR TS=Malawi* OR TS=Malaysia* OR TS=Maldives OR TS=Mali OR TS=“Marshall Island*” OR TS=Mauritania* OR TS=Mexico OR TS=Mexican* OR TS=Micronesia* OR TS=Moldova* OR TS=Mongolia* OR TS=Montenegr* OR TS=Morocc* OR TS=Mozambique OR TS=Myanmar OR TS=Burmese* OR TS=Burma OR TS=Namibia* OR TS=Nepal* OR TS=Nicaragua* OR TS=Niger* OR TS=Niue OR TS=Pakistan* OR TS=Paraguay* OR TS=Peru* OR TS=Philippin* OR TS=Rwanda* OR TS=“Sao Tome” OR TS=Principe OR TS=Senegal* OR TS=Serbia* OR TS=“Sierra Leone*” OR TS=“Solomon Island*” OR TS=Somalia* OR TS=“South Africa*” OR TS=“Sri Lanka” OR TS=“St Lucia” OR TS=“Saint Lucia” OR TS=“St Vincent” OR TS=“Saint Vincent” OR TS=Grenad* OR TS=Sudan* OR TS=Suriname* OR TS=Swaziland* OR TS=Eswatini* OR TS=Syria* OR TS=Tajik* OR TS=Tanzania* OR TS=Zanzibar OR TS=Thai* OR TS=Timor* OR TS=Togo* OR TS=Tonga* OR TS=Tunisia* OR TS=Turkey OR TS=Turkish OR TS=Turkmen* OR TS=Tuvalu* OR TS=Uganda* OR TS=Ukrain* OR TS=Uzbeki* OR TS=Vanuatu* OR TS=Venezuela* OR TS=Vietnam* OR TS=“Viet nam*” OR TS=“West Bank” OR TS=Gaza* OR TS=Palestin* OR TS=Yemen* OR TS=Zambia* OR TS=Zimbabw* OR TS=“Western Sahara” OR TS=Argentin* OR TS=Russia* |
| (3) Trials | TS=“clinical trial*” OR TS=“randomized controlled trial*” OR TS=“controlled clinical trial*” OR TS=“controlled trial*” OR TS=intervention* OR TS=“randomization” OR TS=random* OR TS=trial* OR TS=“clinical trial protocol” OR TS=“clinical study” OR TS=“drug therapy” |
| (4) Balanced energy and protein supplements | (TS=“protein energy” OR TS=“energy protein” OR TS=protein* OR TS=energy*) AND (TS=“supplementation” OR TS=“vitamin supplementation” OR TS=“dietary supplement” OR TS=“food assistance” OR TS=supplement* OR TS=supplementation*) |
| (5) Animal studies | TS=“animal” NOT (TS=“animal” AND TS=“human”) |
| Search strategy | (1) And (2) And (3) And (4) Not (5) |
