# Supplementary material for: The effect of prenatal balanced energy and protein supplementation on small vulnerable newborn types in low- and middle-income countries: A systematic review and meta-analysis of individual participant data
Source: PLoS Med. 2026 Feb 17;23(2):e1004716. doi: 10.1371/journal.pmed.1004716 (PMC12912696; doi:10.1371/journal.pmed.1004716)
Supplement: S4 Table — (DOCX) [file pmed.1004716.s005.docx]

# **S4 Table.** The Cochrane search strategy for identifying randomized controlled trials of prenatal balanced energy and protein supplementation among pregnant women in low- and middle-income countries

| Concept | Cochrane Library Search terms |
| --- | --- |
| (1) Pregnancy | #1 MeSH descriptor: [Pregnancy] explode all trees #2 (Pregnanc*):ti,ab,kw #3 (Pregnant):ti,ab,kw #4 (prenatal):ti,ab,kw #5 (gestation*):ti,ab,kw #6 (antenatal):ti,ab,kw #7 MeSH descriptor: [Pregnant Women] explode all trees #8 (gravid):ti,ab,kw #9 (obstetric):ti,ab,kw #10 (antepartum):ti,ab,kw #11 OR #1-#10 |
| (2) Low- and middle-income countries | #12 MeSH descriptor: [Developing Countries] explode all trees #13 (“developing countr*”):ti,ab,kw #14 (“developing nation*”):ti,ab,kw #15 (“less developed countr*”):ti,ab,kw #16 (“less developed nation*”):ti,ab,kw #17 (“third world nation*”):ti,ab,kw #18 (“third world countr*”):ti,ab,kw #19 (“under developed nation*”):ti,ab,kw #20 (“underdeveloped nation*”):ti,ab,kw #21 (“under developed countr*”):ti,ab,kw #22 (“underdeveloped countr*”):ti,ab,kw #23 (“middle income countr*”):ti,ab,kw #24 (“middle-income countr*”):ti,ab,kw #25 (“middle income nation*”):ti,ab,kw #26 (“middle-income nation*”):ti,ab,kw #27 (“low income countr*”):ti,ab,kw #28 (“low-income countr*”):ti,ab,kw #29 (“low income nation*”):ti,ab,kw #30 (“low-income nation*”):ti,ab,kw #31 (“poor countr*”):ti,ab,kw #32 (“poor nation*”):ti,ab,kw #33 (lmic):ti,ab,kw #34 (lmics):ti,ab,kw #35 MeSH descriptor: [Africa] explode all trees #36 MeSH descriptor: [Asia] explode all trees #37 MeSH descriptor: [South America] explode all trees #38 MeSH descriptor: [Latin America] explode all trees #39 MeSH descriptor: [Central America] explode all trees #40 (africa):ti,ab,kw #41 (asia):ti,ab,kw #42 (“south america*”):ti,ab,kw #43 (“latin america*”):ti,ab,kw #44 (“central america*”):ti,ab,kw #45 (Afghanistan*):ti,ab,kw #46 (Albania*):ti,ab,kw #47 (Algeria*):ti,ab,kw #48 (Samoa*):ti,ab,kw #49 (Angola*):ti,ab,kw #50 (Armenia*):ti,ab,kw #51 (Azerbaijan*):ti,ab,kw #52 (Bangladesh*):ti,ab,kw #53 (Bengali):ti,ab,kw #54 (Belarus*):ti,ab,kw #55 (Belize):ti,ab,kw #56 (Benin):ti,ab,kw #57 (Bhutan*):ti,ab,kw #58 (Bolivia*):ti,ab,kw #59 (Bosnia*):ti,ab,kw #60 (Herzegovina*):ti,ab,kw #61 (Botswana*):ti,ab,kw #62 (Brazil*):ti,ab,kw #63 (Bulgaria*):ti,ab,kw #64 (“Burkina Faso”):ti,ab,kw #65 (Burkinabe):ti,ab,kw #66 (Burundi*):ti,ab,kw #67 (“Cabo Verd*”):ti,ab,kw #68 (“Cape Verd*”):ti,ab,kw #69 (Cambodia*):ti,ab,kw #70 (Cameroon*):ti,ab,kw #71 (“Central African*”):ti,ab,kw #72 (Chad*):ti,ab,kw #73 (China):ti,ab,kw #74 (Chinese):ti,ab,kw #75 (Colombia*):ti,ab,kw #76 (Comoros):ti,ab,kw #77 (Congo):ti,ab,kw #78 (“Costa Rica*”):ti,ab,kw #79 (“Cote d'Ivoire”):ti,ab,kw #80 (“Ivory Coast”):ti,ab,kw #81 (Cuba):ti,ab,kw #82 (Cuban):ti,ab,kw #83 (Djibouti):ti,ab,kw #84 (Dominica*):ti,ab,kw #85 (Ecuador):ti,ab,kw #86 (Egypt*):ti,ab,kw #87 (“El Salvador*”):ti,ab,kw #88 (Eritrea*):ti,ab,kw #89 (Ethiopia*):ti,ab,kw #90 (Fiji*):ti,ab,kw #91 (Gabon*):ti,ab,kw #92 (Gambia*):ti,ab,kw #93 (Georgia*):ti,ab,kw #94 (Ghana*):ti,ab,kw #95 (Grenada*):ti,ab,kw #96 (Guatemala*):ti,ab,kw #97 (Guinea*):ti,ab,kw #98 (Guyan*):ti,ab,kw #99 (Haiti*):ti,ab,kw #100 (Hondura*):ti,ab,kw #101 (India):ti,ab,kw #102 (Indian*):ti,ab,kw #103 (Indonesia*):ti,ab,kw #104 (Iran*):ti,ab,kw #105 (Iraq*):ti,ab,kw #106 (Jamaica*):ti,ab,kw #107 (Jordan*):ti,ab,kw #108 (Kazakh*):ti,ab,kw #109 (Kenya*):ti,ab,kw #110 (Kiribati):ti,ab,kw #111 (“People's Republic of Korea”):ti,ab,kw #112 (“North Korea”):ti,ab,kw #113 (Kosovo):ti,ab,kw #114 (Kosovar*):ti,ab,kw #115 (Kyrgyz*):ti,ab,kw #116 (Lao):ti,ab,kw #117 (Laos):ti,ab,kw #118 (Laotian*):ti,ab,kw #119 (Lebanon):ti,ab,kw #120 (Lebanes*):ti,ab,kw #121 (Lesotho):ti,ab,kw #122 (Liberia*):ti,ab,kw #123 (Libya*):ti,ab,kw #124 (Macedonia*):ti,ab,kw #125 (Madagascar*):ti,ab,kw #126 (Malawi*):ti,ab,kw #127 (Malaysia*):ti,ab,kw #128 (Maldives):ti,ab,kw #129 (Mali):ti,ab,kw #130 (“Marshall Island*”):ti,ab,kw #131 (Mauritania*):ti,ab,kw #132 MeSH descriptor: [Mexico] explode all trees #133 (Mexico):ti,ab,kw #134 (Mexican*):ti,ab,kw #135 (Micronesia*):ti,ab,kw #136 (Moldova*):ti,ab,kw #137 (Mongolia*):ti,ab,kw #138 (Montenegr*):ti,ab,kw #139 (Morocc*):ti,ab,kw #140 (Mozambique):ti,ab,kw #141 (Myanmar):ti,ab,kw #142 (Burmese*):ti,ab,kw #143 (Burma):ti,ab,kw #144 (Namibia*):ti,ab,kw #145 (Nepal*):ti,ab,kw #146 (Nicaragua*):ti,ab,kw #147 (Niger*):ti,ab,kw #148 (Niue):ti,ab,kw #149 (Pakistan*):ti,ab,kw #150 (Paraguay*):ti,ab,kw #151 (Peru*):ti,ab,kw #152 (Philippin*):ti,ab,kw #153 (Rwanda*):ti,ab,kw #154 (“Sao Tome”):ti,ab,kw #155 (Principe):ti,ab,kw #156 (Senegal*):ti,ab,kw #157 (Serbia*):ti,ab,kw #158 (“Sierra Leone*”):ti,ab,kw #159 (“Solomon Island*”):ti,ab,kw #160 (Somalia*):ti,ab,kw #161 (“South Africa*”):ti,ab,kw #162 (“Sri Lanka”):ti,ab,kw #163 (“St Lucia”):ti,ab,kw #164 (“Saint Lucia”):ti,ab,kw #165 (“St Vincent”):ti,ab,kw #166 (“Saint Vincent”):ti,ab,kw #167 (Grenad*):ti,ab,kw #168 (Sudan*):ti,ab,kw #169 (Suriname*):ti,ab,kw #170 (Swaziland*):ti,ab,kw #171 (Eswatini*):ti,ab,kw #172 (Syria*):ti,ab,kw #173 (Tajik*):ti,ab,kw #174 (Tanzania*):ti,ab,kw #175 (Zanzibar):ti,ab,kw #176 (Thai*):ti,ab,kw #177 (Timor*):ti,ab,kw #178 (Togo*):ti,ab,kw #179 (Tonga*):ti,ab,kw #180 (Tunisia*):ti,ab,kw #181 (Turkey):ti,ab,kw #182 (Turkish):ti,ab,kw #183 (Turkmen*):ti,ab,kw #184 (Tuvalu*):ti,ab,kw #185 (Uganda*):ti,ab,kw #186 (Ukrain*):ti,ab,kw #187 (Uzbeki*):ti,ab,kw #188 (Vanuatu*):ti,ab,kw #189 (Venezuela*):ti,ab,kw #190 (Vietnam*):ti,ab,kw #191 (“Viet nam*”):ti,ab,kw #192 (“West Bank”):ti,ab,kw #193 (Gaza*):ti,ab,kw #194 (Palestin*):ti,ab,kw #195 (Yemen*):ti,ab,kw #196 (Zambia*):ti,ab,kw #197 (Zimbabw*):ti,ab,kw #198 (“Western Sahara”):ti,ab,kw #199 (Argentin*):ti,ab,kw #200 (Russia*):ti,ab,kw #201 OR #12-#200 |
| (3) Trials | #224 MeSH descriptor: [Clinical Trials as Topic] explode all trees #225 MeSH descriptor: [Randomized Controlled Trials as Topic] explode all trees #226 MeSH descriptor: [Randomized Controlled Trial] explode all trees #227 (“controlled trial*”):ti,ab,kw #228 (intervention*):ti,ab,kw #229 MeSH descriptor: [Random Allocation] explode all trees #230 (random*):ti,ab,kw #231 (trial*):ti,ab,kw #232 MeSH descriptor: [Clinical Trial Protocol] explode all trees #233 MeSH descriptor: [Clinical Trial Protocols as Topic] explode all trees #234 MeSH descriptor: [Clinical Study] explode all trees #235 MeSH descriptor: [Clinical Studies as Topic] explode all trees #236 MeSH descriptor: [Therapeutic Uses] explode all trees #237 OR #224-#236 |
| (4) Balanced energy and protein supplements | #238 MeSH descriptor: [Energy Intake] explode all trees #239 MeSH descriptor: [Dietary Proteins] explode all trees #240 (“protein energy”):ti,ab,kw #241 (“energy protein”):ti,ab,kw #242 (protein*):ti,ab,kw #243 (energy*):ti,ab,kw #244 OR #238-#243 #245 MeSH descriptor: [Dietary Supplements] explode all trees #246 (supplement*):ti,ab,kw #247 (supplementation*):ti,ab,kw #248 OR #245-#247 #249 #244 AND #248 |
| Search strategy | (1) And (2) And (3) And (4) |
