# Supplementary material for: The effect of prenatal balanced energy and protein supplementation on small vulnerable newborn types in low- and middle-income countries: A systematic review and meta-analysis of individual participant data
Source: PLoS Med. 2026 Feb 17;23(2):e1004716. doi: 10.1371/journal.pmed.1004716 (PMC12912696; doi:10.1371/journal.pmed.1004716)
Supplement: S5 Table — (DOCX) [file pmed.1004716.s006.docx]

# **S5 Table.** Characteristics of the 18 studies identified but not included in the individual participant data meta-analysis of the effect of prenatal balanced energy and protein supplements on small vulnerable newborn types

| Study | Country | Intervention | Comparison | Timing of intervention initiation | Daily energy content | Forms of BEP |
| --- | --- | --- | --- | --- | --- | --- |
| Lechtig 1975^1^ | Guatemala | Protein-rich gruel (*atole*) providing 163 kcal and 11.5 g protein | Nonprotein, low-energy drink (*fresco*) providing 59 kcal | During pregnancy | 163 kcal | Food rations |
| Mora 1979^2^ | Colombia | 60 g dried skim milk, 150 g enriched bread, and 20 g vegetable oil beginning in the third trimester. The supplement provided 856 kcal and 38.4 g protein | Normal, non-supplemented diet | From 6 months of pregnancy | 856 kcal | Food rations |
| Girija 1984^3^ | India | 50 g of sesame cake, 40 g of jaggery, and 10 g of oil. The supplement contributed 30 g protein and 417 kcal | No intervention | From the third trimester of pregnancy | 417 kcal | Food rations |
| Ross 1985^4^ | South Africa | Group 3 received a high bulk supplement, a mixture of beans and maize in a 1.2:1 ratio as mush with added vitamins. The supplement in Group 3 provided 776 kcal and 36 g vegetable protein. Group 4 received a low bulk supplementation, a porridge containing 100 g dry skimmed milk, maize flour, vitamins, and minerals. The supplement in Group 4 provided 700 kcal, 8 g vegetable protein, and 36 g animal protein. | Group 1 received placebo pills, and Group 2 received 30-90 mg of zinc gluconate daily | From 20 weeks gestation to delivery | 776 kcal (high bulk supplement); 700 kcal (low bulk supplement) | Food rations |
| Kardjati 1988^5^ | Indonesia | High energy beverage providing 465 kcal, 7.1 g protein, 50% fat, 10% casein, and 40% glucose, in 75 g dry mix per day | Low energy beverage providing 52 kcal, 6.2 g protein, 50% casein, 28% glucose, and 22% Textaid, in 15 g dry mix per day | From 26 to 28 weeks gestation | 465 kcal | Beverage |
| Ceesay 1997^6^ | The Gambia | High-energy ground biscuits provided before delivery. The biscuits contained roasted groundnuts, rice flour, sugar, and groundnut oil, and they provide a maximum possible daily intake (two biscuits) of 1016 kcal energy, 22 g protein, 56 g fat, 47 mg calcium, and 1.8 mg iron. | No intervention during pregnancy (the same high-energy ground biscuits provided after delivery) | From around 20 weeks gestation to delivery | 1016 kcal | Food rations |
| Kaseb, 2002^7^ | Iran | Traditional food supplements of 400 kcal and 15 g protein daily. Supplements were composed of rice-milk porridge, lentils, pottage, cheese, yogurt, eggs, and milk with bread, given five days a week. Supplements were delivered from the fourth month of pregnancy to childbirth | No supplementation | From 4 months of gestation | 400 kcal | Food ration |
| Potdar 2014^8^ | India | A daily snack made from green leafy vegetables, fruit, and milk provided from $\geq$90 d before pregnancy until delivery in addition to the usual diet. The snacks contained 165 kcal of energy and 10–23% of WHO Reference Nutrient Intakes of b-carotene, riboflavin, folate, vitamin B-12, calcium, and iron | A daily snack made from low-micronutrient vegetables (potato and onion) provided from $\geq$90 d before pregnancy until delivery in addition to the usual diet. The snacks contained 88 kcal and 0–7% of WHO Reference Nutrient Intakes of b-carotene, riboflavin, folate, vitamin B-12, calcium, and iron | ≥ 3 months before pregnancy | 165 kcal | Food rations |
| Dwarkanath 2016^9^ | India | A daily dietary supplement of 300 kcal and 15 g protein, provided as three small, round granola-type treats (*ladoos*), and made of crushed roasted peanuts, puffed rice, skimmed milk, clarified butter, and unrefined sugar. The supplement was provided from the first trimester of pregnancy to delivery | Habitual diet with no supplement | From 12 $\pm$ 1 weeks gestation to delivery | 300 kcal | Food rations |
| Devi 2017^10^ | India | 1. 500 mL of milk/d plus a 10-mg vitamin B-12 tablet/d  2. Milk of 500 mL/d plus a placebo tablet.  The 500 mL milk supplement in both arms provided 320 kcal energy and 16.5 g protein, equivalent to a 21% protein-energy ratio | A placebo tablet only | From 11 $\pm$ 2 weeks gestation to delivery | 320 kcal | Beverage |
| Mantaring 2018^11^ | Philippines | A beverage supplement providing 140 kcal and 7.9 g protein per serving, multivitamin/ minerals, enriched or not with the probiotics *Lactobacillus rhamnosus* and *Bifidobacterium lactis*, from the third trimester of pregnancy until at least two months post-delivery | No intervention | From 24-28 weeks of gestation to 2 months after delivery | 140 kcal | Beverage |
| Olney 2018^12^ | Guatemala | Permutation of varying family ration sizes and individual ration types: 1) full family ration (rice, beans, oil) + CSB; 2) reduced family ration + CSB; 3) no family ration + CSB; 4) full family ration + LNS; 5) full family ration + MNP | No intervention | From pregnancy to up to 6 months postpartum | 270 kcal/d per capita from family ration; 118 kcal from LNS | Food rations |
| Stevens 2018^13^ | Bangladesh | The supplement consisted of 27% pigeon pea, 35% banana, 16% sugar, 9% peanuts, 6% whole milk powder, 6% sesame seeds and 1% iodized salt. The serving size was 173 g, providing 522 kcal, 19.5 g protein, 15.8 g fat, and various vitamins and minerals | No supplement | During pregnancy | 522 kcal | Food rations |
| Neufeld, 2019^14^ | Mexico | Micronutrient-fortified foods providing 250 kcal and 12 g protein | 1) MMS tablets; 2) micronutrient powder | < 25 weeks gestation | 250 kcal | Food ration |
| Tabrizi 2019^15^ | Iran | The food supplement was given every 2 months and included 10 kg of rice, 1400 g of pasta, 5 kg of frozen chicken, 6 cans of tuna, 4 kg of lentils, 2 kg of soybeans, 2 kg packaged palm, 2 kg packaged cheese, 2.5 kg of oil, 1 kg of honey, and 2 kg of sugar | No supplement | From 10 weeks gestation to delivery | 1500 kcal | Food rations |
| Lee, 2022^16^ | Ethiopia | 1. Enhanced nutrition package with placebo infection control tablet. The enhanced nutrition package included daily MMS; participants with MUAC <23 cm received daily BEP in the form of CSB (SuperCereal) providing 760 kcal and 28 g protein daily  2. Enhanced nutrition package with azithromycin  3. Enhanced nutrition package with enhanced infection management package | 1. Routine care with placebo infection control tablet  2. Routine care with azithromycin  3. Routine care with enhanced infection management package | From < 24 weeks gestation to delivery | 760 kcal | Food rations |
| Erchick, 2023^17^ | Nepal | A ready-to-eat snack in the form of lipid-based peanut paste packaged in individual sachets (72g). Each sachet is a daily portion that provides calories (~400 kcal), protein (~14g), and multiple micronutrients at the estimated average requirement for pregnancy | Recommendation to enroll in ANC at a local health clinic and deliver at a certified birthing facility; nutrition, hygiene, breastfeeding, and infant care counseling; and a clean birthing kit. In pregnancy, women in both arms received IFA and albendazole if not provided via ANC | From 14 weeks gestation through the first 6 months of lactation | 400 kcal | Lipid-based supplement |
| NCT03558464 | Kenya | LNS (1 sachet/day), anthelminthic treatment (mebendazole once during the second trimester), soap and chlorine solution for the woman and the household, and agricultural training | Agricultural training | From ≤ 20 weeks gestation to up to 6 months postpartum | Unclear | Lipid-based supplement |

^1^ BEP, balanced energy and protein; CSB, corn-soy blend; GA, gestational age; GWG, gestational weight gain; IFA, iron and folic acid; IOM, Institute of Medicine; LMP, last menstrual period; LNS, lipid-based nutrient supplements; MMN, multiple micronutrients; MMS, multiple micronutrient supplements; MNP, micronutrient powder; MUAC, mid-upper arm circumference; PLA, participatory learning and action; UNIMMAP, UNICEF/WHO/United Nations multiple micronutrient supplements for pregnant and lactating women; WaSH, water, sanitation and hygiene; WHO, World Health Organization.

# **References**

1. Lechtig A, Habicht JP, Delgado H, Klein RE, Yarbrough C, Martorell R. Effect of food supplementation during pregnancy on birthweight. *Pediatrics* 1975; **56**(4): 508-20.

2. Mora JO, de Paredes B, Wagner M, et al. Nutritional supplementation and the outcome of pregnancy. I. Birth weight. *Am J Clin Nutr* 1979; **32**(2): 455-62.

3. Girija A, Geervani P, Rao GN. Influence of dietary supplementation during pregnancy on lactation performance. *J Trop Pediatr* 1984; **30**(2): 79-83.

4. Ross SM, Nel E, Naeye RL. Differing effects of low and high bulk maternal dietary supplements during pregnancy. *Early Hum Dev* 1985; **10**(3-4): 295-302.

5. Kardjati S, Kusin JA, De With C. Energy supplementation in the last trimester of pregnancy in East Java: I. Effect on birthweight. *Br J Obstet Gynaecol* 1988; **95**(8): 783-94.

6. Ceesay SM, Prentice AM, Cole TJ, et al. Effects on birth weight and perinatal mortality of maternal dietary supplements in rural Gambia: 5 year randomised controlled trial. *Bmj* 1997; **315**(7111): 786-90.

7. Kaseb F, Kimiagar M, Ghafarpoor M, Valaii N. Effect of traditional food supplementation during pregnancy on maternal weight gain and birthweight. *Int J Vitam Nutr Res* 2002; **72**(6): 389-93.

8. Potdar RD, Sahariah SA, Gandhi M, et al. Improving women's diet quality preconceptionally and during gestation: effects on birth weight and prevalence of low birth weight--a randomized controlled efficacy trial in India (Mumbai Maternal Nutrition Project). *Am J Clin Nutr* 2014; **100**(5): 1257-68.

9. Dwarkanath P, Hsu JW, Tang GJ, et al. Energy and Protein Supplementation Does Not Affect Protein and Amino Acid Kinetics or Pregnancy Outcomes in Underweight Indian Women. *J Nutr* 2016; **146**(2): 218-26.

10. Devi S, Mukhopadhyay A, Dwarkanath P, et al. Combined Vitamin B-12 and Balanced Protein-Energy Supplementation Affect Homocysteine Remethylation in the Methionine Cycle in Pregnant South Indian Women of Low Vitamin B-12 Status. *J Nutr* 2017; **147**(6): 1094-103.

11. Mantaring J, Benyacoub J, Destura R, et al. Effect of maternal supplement beverage with and without probiotics during pregnancy and lactation on maternal and infant health: a randomized controlled trial in the Philippines. *BMC Pregnancy Childbirth* 2018; **18**(1): 193.

12. Olney DK, Leroy J, Bliznashka L, Ruel MT. PROCOMIDA, a Food-Assisted Maternal and Child Health and Nutrition Program, Reduces Child Stunting in Guatemala: A Cluster-Randomized Controlled Intervention Trial. *J Nutr* 2018; **148**(9): 1493-505.

13. Stevens B, Watt K, Brimbecombe J, Clough A, Judd JA, Lindsay D. A village-matched evaluation of providing a local supplemental food during pregnancy in rural Bangladesh: a preliminary study. *BMC Pregnancy Childbirth* 2018; **18**(1): 286.

14. Neufeld LM, García-Guerra A, Quezada AD, et al. A Fortified Food Can Be Replaced by Micronutrient Supplements for Distribution in a Mexican Social Protection Program Based on Results of a Cluster-Randomized Trial and Costing Analysis. *J Nutr* 2019; **149**(Suppl 1): 2302s-9s.

15. Tabrizi JS, Asghari A, Pourali F, Kousha H, Nikniaz L. Effects of Food Supplementation During Pregnancy on Maternal Weight Gain, Hemoglobin Levels and Pregnancy Outcomes in Iran. *Matern Child Health J* 2019; **23**(2): 258-64.

16. Lee AC, Abate FW, Mullany LC, et al. Enhancing Nutrition and Antenatal Infection Treatment (ENAT) study: protocol of a pragmatic clinical effectiveness study to improve birth outcomes in Ethiopia. *BMJ Paediatr Open* 2022; **6**(1).

17. Erchick DJ, Lama TP, Khatry SK, et al. Supplementation with fortified balanced energy-protein during pregnancy and lactation and its effects on birth outcomes and infant growth in southern Nepal: protocol of a 2×2 factorial randomised trial. *BMJ Paediatr Open* 2023; **7**(1).
