# Supplementary material for: The effect of prenatal balanced energy and protein supplementation on small vulnerable newborn types in low- and middle-income countries: A systematic review and meta-analysis of individual participant data
Source: PLoS Med. 2026 Feb 17;23(2):e1004716. doi: 10.1371/journal.pmed.1004716 (PMC12912696; doi:10.1371/journal.pmed.1004716)
Supplement: S6 Table — (DOCX) [file pmed.1004716.s007.docx]

**S6 Table.** Missingness in outcome data in the included trials on balanced energy and protein supplements^1^

| Study | Total sample size | Missing GA at birth^2^ | Missing birthweight | Missing infant sex | Missing GA at birth, birthweight, or infant sex | Sample size in analysis |
| --- | --- | --- | --- | --- | --- | --- |
| Huybregts, 2009 | 1298 | 94 (7.2) | 219 (16.9) | 196 (15.1) | 257 (19.8) | 1041 |
| Moore, 2012 | 869 | 74 (8.5) | 183 (21.1) | 50 (5.8) | 203 (23.4) | 666 |
| Saville, 2018 | 6004 | 398 (6.6) | 3624 (60.4) | 905 (15.1) | 3930 (65.5) | 2074 |
| Hambidge, 2019 | 1370 | 83 (6.1) | 65 (4.7) | 1 (0.1) | 137 (10.0) | 1233 |
| Khan, 2021 | 2030 | 254 (12.5) | 1978 (97.4) | 390 (19.2) | 1984 (97.7) | 46 |
| Taneja, 2022 | 2222 | 62 (2.8) | 190 (8.6) | 37 (1.7) | 213 (9.6) | 2009 |
| de Kok, 2022 | 2016 | 181 (9.0) | 299 (14.8) | 274 (13.6) | 469 (23.3) | 1547 |
| Muhammad, 2022 | 1884 | 167 (8.9) | 157 (8.3) | 95 (5.0) | 248 (13.2) | 1636 |
| Total | 17693 | 1313 (7.4) | 6715 (38.0) | 1948 (11.0) | 7441 (42.1) | 10252 |

^1^ The values are counts and percentages by study and by analysis. GA, gestational age.

^2^ Including gestational age at birth less than 168 days or greater than 300 days, for which birthweight for sex and gestational age based on the INTERGROWTH-21^st^ newborn size standards could not be derived.
