# Supplementary material for: The effect of prenatal balanced energy and protein supplementation on small vulnerable newborn types in low- and middle-income countries: A systematic review and meta-analysis of individual participant data
Source: PLoS Med. 2026 Feb 17;23(2):e1004716. doi: 10.1371/journal.pmed.1004716 (PMC12912696; doi:10.1371/journal.pmed.1004716)
Supplement: S7 Table — (DOCX) [file pmed.1004716.s008.docx]

**S7 Table.** Effects of prenatal balanced energy and protein supplements on newborn types based on the four-group categorization by maternal characteristics as potential effect modifiers^1^

|  | **Newborn types based on the four-group categorization** | | | | | | | | |
| --- | --- | --- | --- | --- | --- | --- | --- | --- | --- |
|  | **Term-SGA** | | | **Preterm-nonSGA** | | | **Preterm-SGA** | | |
|  | **Number of studies** | **Pooled RR (95% CI)** | ***P* for interaction** | **Number of studies** | **Pooled RR (95% CI)** | ***P* for interaction** | **Number of studies** | **Pooled RR (95% CI)** | ***P* for interaction** |
| Maternal age, years |  |  |  |  |  |  |  |  |  |
| < 20 | 7 | 1.03 (0.89, 1.18) | 0.13 | 6 | 1.21 (0.78, 1.86) | 0.25 | 6 | 1.10 (0.68, 1.78) | 0.11 |
| 20-29 | 8 | 0.85 (0.76, 0.96) |  | 7 | 0.83 (0.73, 0.94) |  | 5 | 0.71 (0.33, 1.53) |  |
| ≥ 30 | 8 | 0.94 (0.81, 1.09) |  | 7 | 0.92 (0.64, 1.32) |  | 3 | 0.36 (0.14, 0.94) |  |
| Parity |  |  |  |  |  |  |  |  |  |
| 0 | 7 | 1.00 (0.89, 1.12) | 0.19 | 6 | 1.18 (0.92, 1.51) | 0.099 | 4 | 1.28 (0.70, 2.32) | 0.026 |
| ≥ 1 | 7 | 0.90 (0.82, 0.99) |  | 7 | 0.94 (0.85, 1.05) |  | 5 | 0.53 (0.32, 0.87) |  |
| Gestational age at enrollment, weeks |  |  |  |  |  |  |  |  |  |
| < 20 | 7 | 0.88 (0.81, 0.96) | 0.03 | 8 | 0.87 (0.76, 0.98) | 0.05 | 6 | 0.69 (0.57, 0.85) | 0.01 |
| ≥ 20 | 7 | 1.12 (0.92, 1.36) |  | 4 | 1.55 (0.87, 2.76) |  | 1 | 6.85 (1.21, 38.72) |  |
| Early-pregnancy BMI, kg/m^2^ |  |  |  |  |  |  |  |  |  |
| < 18.5 | 8 | 0.84 (0.77, 0.92) | 0.54 | 8 | 0.81 (0.67, 0.98) | 0.52 | 3 | 0.64 (0.41, 0.98) | 0.97 |
| 18.5 to < 25.0 | 7 | 0.90 (0.81, 0.99) |  | 7 | 0.90 (0.81, 1.00) |  | 6 | 0.61 (0.34, 1.08) |  |
| ≥ 25.0 | 7 | 0.82 (0.71, 0.94) |  | 5 | 0.95 (0.76, 1.19) |  | 1 | 0.48 (0.04, 5.28) |  |
| Maternal anemia |  |  |  |  |  |  |  |  |  |
| No anemia | 5 | 0.87 (0.74, 1.02) | 0.55 | 5 | 0.82 (0.61, 1.10) | 0.69 | 4 | 0.33 (0.15, 0.73) | 0.026 |
| Mild anemia | 6 | 0.86 (0.64, 1.15) |  | 5 | 0.91 (0.73, 1.13) |  | 4 | 1.08 (0.78, 1.49) |  |
| Moderate to severe anemia | 5 | 1.00 (0.81, 1.23) |  | 5 | 0.96 (0.79, 1.16) |  | 3 | 1.03 (0.35, 3.02) |  |

^1^ Values are pooled risk ratios and 95% confidence intervals from random-effects meta-analytical models comparing prenatal balanced energy and protein supplements with control. The study-specific estimates (omitted from the table for brevity) were calculated using log-binomial or modified Poisson models. Term-nonSGA was used as the reference group in all models. BMI, body mass index; CI, confidence interval; nonSGA, not small for gestational age; RR, risk ratio; SGA, small for gestational age.
