# Supplementary material for: The effect of prenatal balanced energy and protein supplementation on small vulnerable newborn types in low- and middle-income countries: A systematic review and meta-analysis of individual participant data
Source: PLoS Med. 2026 Feb 17;23(2):e1004716. doi: 10.1371/journal.pmed.1004716 (PMC12912696; doi:10.1371/journal.pmed.1004716)
Supplement: S8 Table — (DOCX) [file pmed.1004716.s009.docx]

**S8 Table.** Effects of prenatal balanced energy and protein supplements on newborn types based on the four-group categorization by study-level characteristics^1^

|  | **Newborn types based on the four-group categorization** | | | | | | | | |
| --- | --- | --- | --- | --- | --- | --- | --- | --- | --- |
|  | **Term-SGA** | | | **Preterm-nonSGA** | | | **Preterm-SGA** | | |
|  | **Number of studies** | **Pooled RR (95% CI)** | ***P* for interaction** | **Number of studies** | **Pooled RR (95% CI)** | ***P* for interaction** | **Number of studies** | **Pooled RR (95% CI)** | ***P* for interaction** |
| Energy content of BEP supplements, kcal/d |  |  |  |  |  |  |  |  |  |
| 250 - < 500 | 4 | 0.84 (0.74, 0.94) | 0.13 | 4 | 0.86 (0.69, 1.06) | 0.29 | 4 | 0.62 (0.42, 0.91) | 0.26 |
| 500 - < 1000 | 4 | 0.97 (0.84, 1.11) |  | 4 | 0.98 (0.86, 1.13) |  | 2 | 0.93 (0.52, 1.67) |  |
| Percent energy from protein |  |  |  |  |  |  |  |  |  |
| 10% to < 15% | 3 | 0.90 (0.83, 0.97) | 0.58 | 3 | 0.92 (0.80, 1.07) | 0.95 | 2 | 0.75 (0.60, 0.95) | 0.54 |
| 15% to 20% | 5 | 0.85 (0.71, 1.02) |  | 5 | 0.91 (0.73, 1.14) |  | 4 | 0.63 (0.36, 1.08) |  |
| Forms of BEP |  |  |  |  |  |  |  |  |  |
| Food ration | 3 | 0.80 (0.57, 1.13) | 0.58 | 3 | 0.81 (0.48, 1.36) | 0.60 | 2 | 0.61 (0.21, 1.79) | 0.71 |
| Lipid-based supplement | 5 | 0.89 (0.87, 0.90) |  | 5 | 0.93 (0.84, 1.02) |  | 4 | 0.75 (0.60, 0.93) |  |
| Control group |  |  |  |  |  |  |  |  |  |
| IFA^2^ | 6 | 0.86 (0.77, 0.95) | 0.17 | 6 | 0.88 (0.75, 1.02) | 0.15 | 5 | 0.69 (0.49, 0.96) | 0.91 |
| MMS | 2 | 0.98 (0.84, 1.15) |  | 2 | 1.12 (0.83, 1.50) |  | 1 | 0.65 (0.23, 1.79) |  |

^1^ Values are pooled risk ratios and 95% confidence intervals from random-effects meta-analytical models comparing prenatal balanced energy and protein supplements with control. The study-specific estimates (omitted from the table for brevity) were calculated using log-binomial or modified Poisson models. Term-nonSGA was used as the reference group in all models. BMI, body mass index; CI, confidence interval; IFA, iron and folic acid supplements; MMS, multiple micronutrient supplements; nonSGA, not small for gestational age; RR, risk ratio; SGA, small for gestational age.

^2^ Included studies in which standard antenatal care was the control group.
