# Supplementary material for: The effect of prenatal balanced energy and protein supplementation on small vulnerable newborn types in low- and middle-income countries: A systematic review and meta-analysis of individual participant data
Source: PLoS Med. 2026 Feb 17;23(2):e1004716. doi: 10.1371/journal.pmed.1004716 (PMC12912696; doi:10.1371/journal.pmed.1004716)
Supplement: S10 Table — (DOCX) [file pmed.1004716.s011.docx]

**S10 Table.** Effects of prenatal balanced energy and protein supplements on newborn types based on the four-group categorization when restricting to studies with ultrasound-based measures of gestational age^1^

|  | **Newborn types based on the four-group categorization** | | | |
| --- | --- | --- | --- | --- |
|  | **Term-nonSGA** | **Term-SGA** | **Preterm-nonSGA** | **Preterm-SGA** |
|  | **RR (95% CI)** | **RR (95% CI)** | **RR (95% CI)** | **RR (95% CI)** |
| Huybregts, 2009 | Reference | 0.90 (0.72, 1.13) | 1.15 (0.85, 1.56) | 0.65 (0.23, 1.79) |
| Moore, 2012 | Reference | 1.06 (0.85, 1.31) | 0.67 (0.19, 2.34) | Low events (*n* = 1) |
| Hambidge, 2019 | Reference | 0.88 (0.87, 0.90) | 0.81 (0.54, 1.20) | 0.75 (0.59, 0.95) |
| Taneja, 2022 | Reference | 0.71 (0.62, 0.81) | 0.65 (0.50, 0.84) | 0.37 (0.21, 0.66) |
| de Kok, 2022 | Reference | 0.87 (0.71, 1.07) | 0.89 (0.78, 1.02) | 0.82 (0.28, 2.41) |
| Muhammad, 2022 | Reference | 0.87 (0.72, 1.05) | 0.95 (0.81, 1.11) | 0.79 (0.35, 1.77) |
| Pooled, fixed-effect | Reference | 0.88 (0.87, 0.90) | 0.89 (0.81, 0.97) | 0.69 (0.56, 0.84) |
| Pooled, random-effects | Reference | 0.86 (0.79, 0.95) | 0.88 (0.76, 1.01) | 0.65 (0.49, 0.88) |

^1^ Values are risk ratios and 95% confidence intervals comparing prenatal balanced energy and protein supplements with control, computed using log-binomial or modified Poisson models. Estimates are not available for models marked with low events (with the number of outcome events shown in parentheses) due to failure of model convergence. CI, confidence interval; nonSGA, not small for gestational age; RR, risk ratio; SGA, small for gestational age.
