# Supplementary material for: The effect of prenatal balanced energy and protein supplementation on small vulnerable newborn types in low- and middle-income countries: A systematic review and meta-analysis of individual participant data
Source: PLoS Med. 2026 Feb 17;23(2):e1004716. doi: 10.1371/journal.pmed.1004716 (PMC12912696; doi:10.1371/journal.pmed.1004716)
Supplement: S11 Table — (DOCX) [file pmed.1004716.s012.docx]

**S11 Table**. Comparison of pooled random-effects estimates with and without using the Hartung-Knapp-Sidik-Jonkman method to adjust the variance estimates for the numbers of studies included in the meta-analyses^1^

| Outcome | Without Hartung-Knapp-Sidik-Jonkman adjustment (primary analysis) | With Hartung-Knapp-Sidik-Jonkman adjustment (sensitivity analysis) |
| --- | --- | --- |
| Term-SGA-nonLBW | 0.90 (0.80, 1.02) | 0.90 (0.78, 1.05) |
| Term-SGA-LBW | 0.80 (0.72, 0.90) | 0.80 (0.69, 0.93) |
| Preterm-LGA-nonLBW | 0.95 (0.78, 1.15) | 0.95 (0.74, 1.22) |
| Preterm-AGA-nonLBW | 0.94 (0.79, 1.11) | 0.94 (0.76, 1.16) |
| Preterm-AGA-LBW | 0.75 (0.60, 0.93) | 0.75 (0.57, 0.98) |
| Term-LGA-nonLBW | 1.17 (0.81, 1.69) | 1.17 (0.73, 1.87) |
| Preterm-SGA-LBW | 0.70 (0.53, 0.91) | 0.70 (0.49, 0.99) |
| Preterm-LGA-LBW | 0.93 (0.50, 1.73) | 0.93 (0.30, 2.89) |
| Term-AGA-LBW | 1.13 (0.57, 2.25) | 1.13 (0.37, 3.46) |
| Term-SGA | 0.88 (0.81, 0.96) | 0.88 (0.78, 0.99) |
| Preterm-nonSGA | 0.91 (0.79, 1.04) | 0.91 (0.76, 1.08) |
| Preterm-SGA | 0.69 (0.52, 0.91) | 0.69 (0.48, 0.99) |

^1^ Values are pooled risk ratios and 95% confidence intervals from random-effects meta-analyses comparing prenatal balanced energy and protein supplements with control. AGA, appropriate for gestational age; LBW, low birthweight; LGA, large for gestational age; BEP, balanced energy and protein; nonLBW, not low birthweight; SGA, small for gestational age.
