# Supplementary material for: The effect of prenatal balanced energy and protein supplementation on small vulnerable newborn types in low- and middle-income countries: A systematic review and meta-analysis of individual participant data
Source: PLoS Med. 2026 Feb 17;23(2):e1004716. doi: 10.1371/journal.pmed.1004716 (PMC12912696; doi:10.1371/journal.pmed.1004716)
Supplement: S12 Table — (DOCX) [file pmed.1004716.s013.docx]

**S12 Table.** Risk of bias of the included studies^1^

|  | Bias arising from the randomization process | Bias due to deviations from intended intervention | Bias due to missing outcome data | Bias in measurement of the outcome | Bias in selection of the reported result | For cluster RCT: bias arising from the timing of identification and recruitment of individual participants in relation to timing of randomization | Overall risk of bias |
| --- | --- | --- | --- | --- | --- | --- | --- |
| Huybregts, 2009 | Low | Low | Low | Low | Low | NA | Low |
| Moore, 2012 | Low | Low | Low | Low | Low | NA | Low |
| Saville, 2018 | Low | Low | Low | Low | Low | Low | Low |
| Hambidge, 2019 | Low | Low | Low | Low | Low | NA | Low |
| Khan, 2021 | Low | Low | Low | Low | Low | Low | Low |
| Taneja, 2022 | Low | Low | Low | Low | Low | NA | Low |
| de Kok, 2022 | Low | Low | Low | Low | Low | NA | Low |
| Muhammad, 2022 | Low | Low | Low | Low | Low | NA | Low |

^1^ RCT, randomized controlled trial.
