# Supplementary material for: The effect of prenatal balanced energy and protein supplementation on small vulnerable newborn types in low- and middle-income countries: A systematic review and meta-analysis of individual participant data
Source: PLoS Med. 2026 Feb 17;23(2):e1004716. doi: 10.1371/journal.pmed.1004716 (PMC12912696; doi:10.1371/journal.pmed.1004716)
Supplement: S1 Fig — (DOCX) [file pmed.1004716.s014.docx]

**
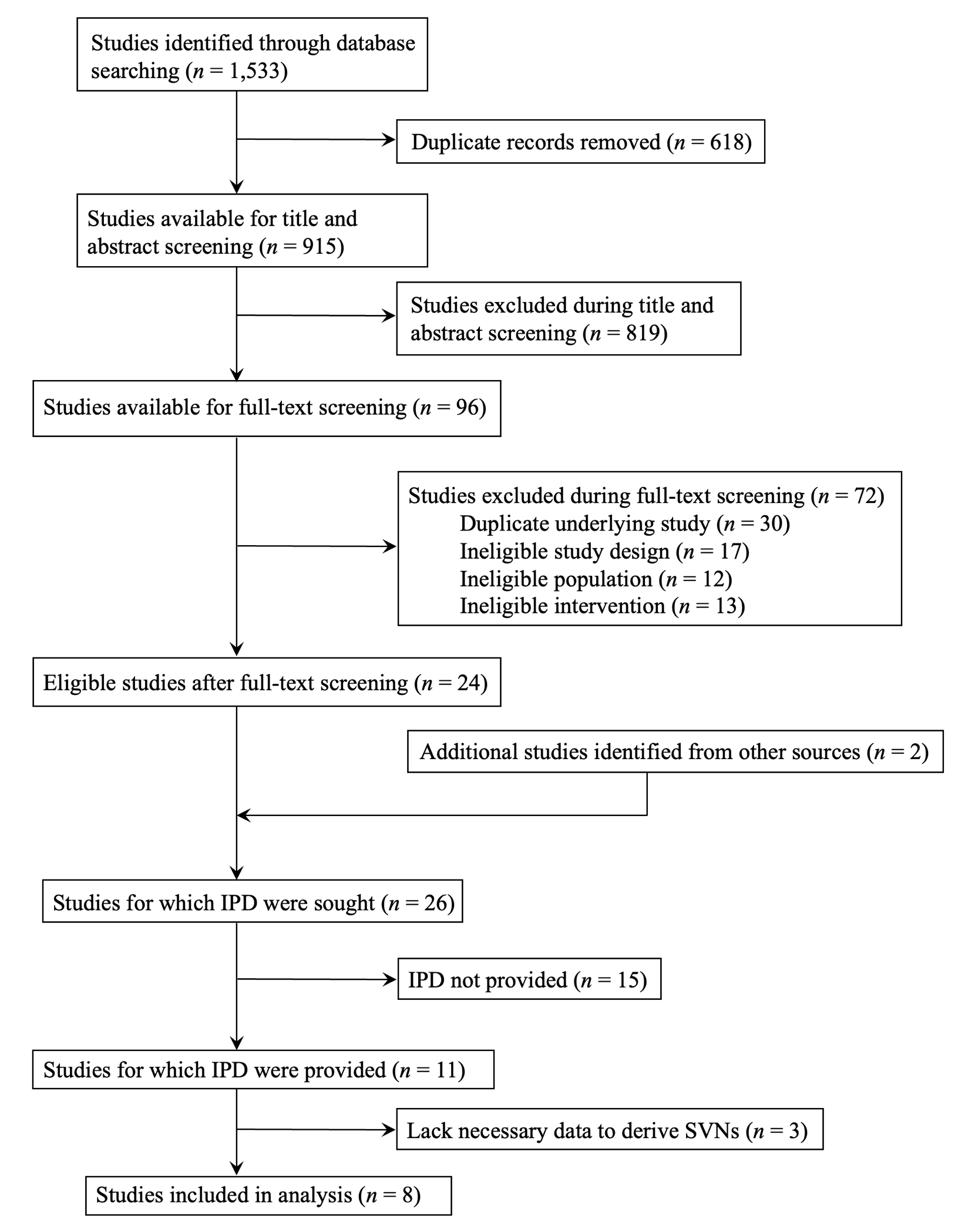
**

**S1 Fig**. PRISMA flow diagram for the individual participant data meta-analysis on the effects of prenatal balanced energy and protein supplements on small vulnerable newborn types in low- and middle-income countries. IPD, individual participant data; SVNs, small vulnerable newborns.
