# Supplementary material for: The effect of prenatal balanced energy and protein supplementation on small vulnerable newborn types in low- and middle-income countries: A systematic review and meta-analysis of individual participant data
Source: PLoS Med. 2026 Feb 17;23(2):e1004716. doi: 10.1371/journal.pmed.1004716 (PMC12912696; doi:10.1371/journal.pmed.1004716)
Supplement: S4 Fig — (DOCX) [file pmed.1004716.s017.docx]

*P*-value from Egger’s test: 0.84

**S4 Fig.** Funnel plot for the effect of prenatal balanced energy and protein supplements on the small vulnerable newborn type of preterm-SGA-LBW. LBW, low birthweight; SGA, small for gestational age.
