# Supplementary material for: The effect of prenatal balanced energy and protein supplementation on small vulnerable newborn types in low- and middle-income countries: A systematic review and meta-analysis of individual participant data
Source: PLoS Med. 2026 Feb 17;23(2):e1004716. doi: 10.1371/journal.pmed.1004716 (PMC12912696; doi:10.1371/journal.pmed.1004716)
Supplement: S6 Fig — (DOCX) [file pmed.1004716.s019.docx]

*P*-value from Egger’s test: 0.34

**S6 Fig.** Funnel plot for the effect of prenatal balanced energy and protein supplements on the small vulnerable newborn type of preterm-AGA-LBW. AGA, appropriate for gestational age; LBW, low birthweight.
