# Supplementary material for: The effect of prenatal balanced energy and protein supplementation on small vulnerable newborn types in low- and middle-income countries: A systematic review and meta-analysis of individual participant data
Source: PLoS Med. 2026 Feb 17;23(2):e1004716. doi: 10.1371/journal.pmed.1004716 (PMC12912696; doi:10.1371/journal.pmed.1004716)
Supplement: S7 Fig — (DOCX) [file pmed.1004716.s020.docx]

*P*-value from Egger’s test: 0.93

**S7 Fig.** Funnel plot for the effect of prenatal balanced energy and protein supplements on the small vulnerable newborn type of term-LGA-nonLBW. LGA, large for gestational age; nonLBW, not low birthweight.
